# Effectiveness of a brief group behavioural intervention on psychological distress in young adolescent Syrian refugees: A randomised controlled trial

**DOI:** 10.1371/journal.pmed.1004046

**Published:** 2022-08-12

**Authors:** Richard A. Bryant, Aiysha Malik, Ibrahim Said Aqel, Maha Ghatasheh, Rand Habashneh, Katie S. Dawson, Sarah Watts, Mark J. D. Jordans, Felicity L. Brown, Mark van Ommeren, Aemal Akhtar

**Affiliations:** 1 School of Psychology, University of New South Wales, Sydney, Australia; 2 Brain Dynamics Centre, Westmead Institute for Medical Research, Sydney, Australia; 3 Department of Mental Health and Substance Abuse, World Health Organization, Geneva, Switzerland; 4 Institute for Family Health, King Hussein Foundation, Amman, Jordan; 5 Research and Development Department, War Child Holland, Amsterdam, the Netherlands; 6 Amsterdam Institute of Social Science Research, University of Amsterdam, Amsterdam, the Netherlands; 7 Department of Clinical, Neuro and Developmental Psychology, World Health Organization Collaborating Centre for Research and Dissemination of Psychological Interventions, Vrije Universiteit, Amsterdam, the Netherlands; Epicentre, FRANCE

## Abstract

**Background:**

Millions of young adolescents in low- and middle-income countries (LMICs) affected by humanitarian crises experience elevated rates of poor mental health. There is a need for scalable programs that can improve the mental health of young adolescents. This study evaluated the effectiveness of a nonspecialist delivered group-based intervention (Early Adolescent Skills for Emotions (EASE)) to improve young adolescents’ mental health.

**Methods and findings:**

In this single-blind, parallel, controlled trial, Syrian refugees aged 10 to 14 years in Jordan were identified through screening of psychological distress as defined by scores ≥15 on the Paediatric Symptom Scale. Participants were randomised to either EASE or enhanced usual care (EUC) involving referral to local psychosocial services (on a 1:1.6 ratio). Participants were aware of treatment allocation but assessors were blinded. Primary outcomes were scores on the Paediatric Symptom Checklist (PSC; internalising, externalising, and attentional difficulty scales) assessed at week 0, 9 weeks, and 3 months after treatment (primary outcome time point). It was hypothesised that EASE would result in greater reductions on internalising symptoms than EUC. Secondary outcomes were depression, posttraumatic stress, well-being, functioning, school belongingness, and caregivers’ parenting and mental health. Between June 2019 and January 2020, 1,842 young adolescent refugees were screened for eligibility on the basis of psychological distress. There were 520 adolescents (28.2%) who screened positive, of whom 471 (90.6%) agreed to enter the trial. Overall, 185 were assigned to EASE and 286 to EUC, and 169 and 254 were retained at 3 months for EASE and EUC, respectively. Intent-to-treat analyses indicated that at 3 months, EASE resulted in greater reduction on the PSC-internalising scale than EUC (estimated mean difference 0.69, 95% CI 0.19 to 1.19; *p* = 0.007; effect size, 0.38) but there were no differences for PSC-externalising (estimated mean difference 0.24, 95% CI −0.43 to 0.91; *p* = 0.49; effect size, −0.10), PSC-attentional problem (estimated mean difference −0.01, 95% CI −0.51 to 0.54; *p* = 0.97; effect size, −0.01) scores, or on depression, posttraumatic stress, well-being, functioning, or school belongingness. Relative to EUC, caregivers in EASE had less psychological distress (estimated mean difference 1.95, 95% CI 0.71 to 3.19; *p* = 0.002) and inconsistent disciplinary parenting (mean difference 1.54, 95% CI 1.03 to 2.05; *p* < 0.001). Secondary analyses that (a) focused on adolescents with probable internalising disorders; (b) completed the 3-month assessment; and (c) controlled for trauma exposure did not alter the primary results. Mediation analysis indicated that for caregivers in the EASE condition, reduction in inconsistent disciplinary parenting was associated with reduced attentional (β = 0.11, SE 0.07; 95% CI 0.003, 0.274) and internalising (β = 0.11, SE 0.07; 95% CI 0.003, 0.274) problems in their children. No adverse events were attributable to the intervention. A limitation was that EUC was not matched to EASE in terms of facilitator attention or group involvement.

**Conclusions:**

EASE led to reduced internalising problems in young refugee adolescents and was associated with reduced distress and less inconsistent disciplinary parenting in caregivers. This intervention has the potential as a scalable intervention to mitigate young adolescents’ emotional difficulties in LMIC.

**Trial registration:**

Prospectively registered at Australian and New Zealand Clinical Trials Registry: ACTRN12619000341123.

## Abstract 2

In a randomized controlled trial, Richard A. Bryant and colleagues study the effectiveness of the Early Adolescent Skills for Emotions (EASE) intervention to improve the mental health of Syrian refugees aged 10-14 years living in Jordan.

## Author summary

**Why was this study done?:**

- There are millions of young adolescents in low- and middle-income countries (LMICs) who are at greater risk for common mental disorders.
- Most LMICs lack sufficient health resources to meet the mental health needs of young adolescents.
- In response to this situation, the World Health Organization (WHO) has developed a brief psychological intervention that can be delivered by trained lay workers that aims to reduce internalising problems in young adolescents.
- This study was the first to test effectiveness of the Early Adolescent Skills for Emotions (EASE) program in distressed young adolescents.

**What did the researchers do and find?:**

- This study screened young adolescent Syrian refugees in Amman, Jordan, and subsequently randomised 471 adolescents who displayed distress to either 7 group sessions of EASE (with 3 group sessions for their caregivers) or enhanced usual care (EUC).
- Independent assessments indicated that 3 months following treatment, adolescents who received EASE reported significantly less internalising symptoms, with a moderate effect relative to EUC.
- EASE also led to greater reductions in caregiver distress and inconsistent disciplinary parenting, and the latter was associated with greater reductions in internalising problems in caregivers’ children.

**What do these findings mean?:**

- This study suggests that internalising problems in young adolescents can be reduced by a brief group program delivered by trained nonspecialists.
- This brief program has the potential to alleviate distress in both the young adolescents and their caregivers.
- LMICs who lack adequate numbers of mental health specialists could benefit from implementing this program to address the mental health needs of young adolescents.

## Introduction

Many humanitarian crises, including wars, disasters, and unrest, occur in low- and middle-income countries (LMICs) where adolescents experience elevated rates of common mental disorders [1]. This presents a critical public health issue as poor mental health during adolescence can be a precursor to persistent mental disorders in adulthood and can also impede attainment of fundamental developmental milestones [2]. There is a dearth of scalable evidence-based programs for this population [3], especially for those who are conflict affected. The Lancet Commission on Global Mental Health and Sustainable Development Goals highlighted the significant gap between mental health needs in poorly resourced countries and the availability of services [4]. A meta-analysis found no reliable evidence of psychological interventions for children or adolescents in reducing anxiety or depression [5]. A recent umbrella review identified 9 meta-analyses of psychosocial interventions for children or adolescents in LMICs and concluded that whereas there was suggestive evidence for treatments of posttraumatic stress disorder (PTSD), there was no evidence for treatment of anxiety or depression [6]. Further, most studies that have been done with adolescents have focused on middle to late adolescents (>15 years of age).

At present, many LMICs cannot scale up interventions because of the scarcity of mental health specialists in LMICs, the tendency for some interventions to be disorder-specific (which requires local health providers to be trained in many protocols), and lengthy interventions that increase costs for local health services and are demanding on service users. The scarcity of mental health specialists in LMICs has led to the adoption of task-shifting approaches in which nonspecialists are trained in the delivery of mental health programs. A meta-analytic review indicates that task-shifting approaches can be effective [7]. The World Health Organization (WHO) has implemented one such program, Problem Management Plus (PM+) [8], a brief 5-session program that adopts a transdiagnostic approach by teaching skills in arousal reduction, problem solving, behavioural activation, and accessing social support [9]. This program has been validated for use with adults in trials in both individual [10] and group [11] formats. There is a significant gap of comparable programs for young adolescents and this has resulted in a huge treatment gap in addressing mental health needs of young adolescents in LMICs.

In response to the need for mental health programs for young adolescents, WHO has developed a brief, transdiagnostic intervention similar to PM+, titled Early Adolescent Skills for Emotions (EASE) that targets 10- to 14-year-old adolescents and aims to reduce internalising problems such as anxiety and depression [12]. This program comprises 7 group sessions for adolescents that focus on arousal reduction, behavioural activation, and problem management as these strategies have been shown to be key for reducing internalising problems in adolescents. The intervention also comprises 3 group sessions for caregivers that teach coping skills, positive parenting, and inform them of the strategies taught to the adolescents.

We conducted a randomised controlled trial (RCT) of EASE with young adolescent Syrian refugees residing in Amman, Jordan and compared this program to enhanced usual care (EUC). The civil war in Syria has resulted in over 13 million Syrians being forcibly displaced. Syrian refugees are at high risk for mental disorders as a result of the war and difficulties with resettling [13]. We hypothesised that adolescents randomised to the intervention would display greater reductions in internalising symptoms relative to EUC. Additionally, we expected that EASE would lead to reduced symptoms of depression, PTSD, functional impairment, and improved wellbeing and school involvement compared to EUC. We also expected that the intervention would lead to greater reductions in psychological distress in caregivers and improved parenting skills relative to EUC, and this would be associated with fewer internalising problems in the adolescents.

## Method

### Study design

This 2-arm, single-blind RCT was conducted in Amman, Jordan. There are an estimated 1.4 million Syrians residing in Jordan [14]. The study was conducted in collaboration with the Institute for Family Health (IFH), a national humanitarian agency. The project was prospectively registered (Australian and New Zealand Clinical Trials Registry, no. ACTRN12619000341123) and was approved locally by the Ethics Committee of Al Basheer Hospital in Jordan, the University of New South Wales Human Research Ethics Committee, and the WHO Ethical Review Committee. The trial protocol is available in the Supporting information (S1 Text). This study is reported as per the Consolidated Standards of Reporting Trials (CONSORT) guideline (S1 Consort Checklist).

### Participants

Participants were recruited by inviting adolescents and their caregivers to participate during door-to-door visits in regions in Amman known to host large numbers of Syrian refugees. Participants were enlisted in the trial if they met the following inclusion criteria: (a) Syrian refugee; (b) aged 10 to 14 years; (c) resided with a related caregiver who could provide legal consent; and (d) scored ≥15 on the Paediatric Symptom Scale (PSC-17) [15] (see Table A in S1 Appendix). The PSC-17 is a 17-item questionnaire that assesses psychological distress in children, with a range of 0 to 34; a cutoff ≥15 has been shown to indicate psychological distress [15]. Exclusion criteria were: (a) unaccompanied minor; (b) minors with an unrelated caregiver; (c) significant developmental, cognitive, or neurological impairments as determined by 4 items from an adapted version of the 10 Questions instrument [16]; or (d) imminent risk of suicide. One child, selected by the caregiver, was recruited per household in order to minimise the burden on the family and reduce likelihood of contamination of the intervention between participants. Any potential participants who met the exclusion criteria were referred to specialised services within IFH or another organization according to Inter-Agency Standard Operating Procedures. Informed consent was obtained from caregivers and assent from the adolescents in 2 stages to participate in (a) the screening; and (b) the EASE trial; participation required written informed consent, except oral consent was accepted for illiterate participants.

### Randomisation and masking

Randomisation was conducted at the University of New South Wales (Australia) by staff who were independent of the trial using computerised software that generated random number sequences. Eligible adolescents were randomised without stratification allocated to either the EASE program or EUC (on a 1:1.6 ratio). Assessors who identified and enrolled participants into the trial and who conducted all assessments were masked to treatment condition allocation. Assessors were managed separately from the rest of the research team and at no point interacted with EASE or EUC facilitators. At the commencement of each postintervention assessment, assessors instructed participants to not inform them about their allocated condition. Further, at each assessment, assessors were instructed to guess which treatment arm the person was assigned to.

Before the trial was conducted, a comprehensive translation and cultural adaptation process of EASE was undertaken for the context of Syrian adolescent refugees in Jordan. Initial adaptation workshops were conducted with Syrian adolescents in Lebanon to agree upon necessary amendments [17], and subsequent adaptation workshops were conducted in Jordan with community representatives and health providers to determine the need for local adaptations. A pilot trial was then conducted to determine feasibility and acceptability of the EASE intervention [18]. All assessment instruments and the EASE protocol were adapted to be acceptable for cultural appropriateness, language, metaphors, and context.

As prescribed by the EASE protocol [12], the intervention comprised 7 weekly 1.5-hour group sessions for adolescents (8 to 10 people per group). In accordance with the findings of the adaptation work, all groups were gender specific and the groups comprised adolescents of any age from 10 to 14 years of age. Session 1 established group rules, provided psychoeducation about the effects of stressful events, and provided strategies on how to identify emotions. Session 2 taught strategies on reducing arousal and focused on relaxation breathing as a core stress management strategy. Sessions 3 and 4 focused on behavioural activation. Sessions 5 and 6 taught problem solving strategies, which included seeking social support as a means of dealing with problems. Session 7 focused on relapse prevention to manage future stressors. One caregiver of each adolescent was invited to three 2-hour group sessions (8 to 10 people per group) that occurred at 2 weekly intervals concurrently with the adolescent sessions. The goal of caregiver sessions was to promote supportive parenting to assist adolescents to manage stress. In session 1, caregivers were provided with psychoeducation and skills to help their child cope when they are distressed. Session 2 promoted positive parenting skills, including increasing praise and reducing physical discipline and promoting communication and relationship skills. Session 3 focused on skills for the caregiver to manage their own stress, including advice about sleep, nutrition, stress reduction exercises, and utilisation of social support. In addition, each session included informing caregivers about the strategies being taught to the adolescents in their sessions (see Table B in S1 Appendix). Sessions were conducted in various locations in Amman to facilitate attendance by participants who received reimbursement for travel costs. A Jordanian supervisor with >15 years in psychosocial programs and who participated in a training of trainer course on the EASE intervention attended 19% of sessions and used a standardised checklist to assess treatment fidelity and facilitator competency (rated on a 4-point scale: 0 = *not satisfactory*, 3 = *very good*).

Each group was conducted by 2 facilitators. The EASE facilitators were from a variety of professional backgrounds and recruited from the community by the implementing partner (IFH) in Jordan specifically to be trained in EASE. Facilitators either had experience implementing psychosocial services and/or tertiary qualification in a variety of disciplines. No facilitators had specialist mental health qualifications, training, or experience. Facilitators received 8 days of training on the EASE protocol, as well as group facilitation skills. Classroom-based competency was assessed through standardised role plays using an adaptation of the ENhancing Assessment of Common Therapeutic (ENACT) framework to assess counsellor competency [19]. Only those who demonstrated competency were permitted to function as facilitators. Facilitators were required to complete 2 closely supervised practice cycles following training. Twenty-three facilitators participated in trainings prior to the start of the trial. Following the completion of the training, all 23 (100%) facilitators demonstrated competency in intervention strategies, basic counselling skills, and group management, and of these, 20 facilitators continued with the current study and conducted EASE groups. Training and weekly supervision was conducted by a local supervisor (MG) who had attended an EASE training of trainer course and was supported biweekly by an experienced lead supervisor (AM) who was involved in developing of EASE protocol.

Participants randomised to EUC received a single 30-minute family session conducted in the participant’s home by a community health worker. This session provided feedback about the adolescent’s responses on the assessment battery, instruction of simple coping strategies, and provided a list of local services that the family could attend for psychosocial support for the adolescent. This was considered enhanced care as there are typically no mental health services accessible for young adolescents in Amman. The EUC facilitators followed a standardised script, and both caregiver and child were invited to attend. Sessions were limited to 30 minutes and provided the family with information on psychological problems, self-care strategies, and local referral options.

### Outcomes

#### Primary outcome

The primary outcome measure was the adolescents’ self-reported responses on the Paediatric Symptom Checklist (PSC-35), a 35-item instrument scored on a 3-point Likert scale (0 = *never*, 2 = *often*) and that has been validated in Middle Eastern settings [20]. The primary subscales index internalising, externalising, attentional, as well as providing a total score of children’s mental health, with higher total scores reflecting more severe problems. The primary outcome included both subscale and total scores because of the goal of EASE to target internalising problems. Recommended cutoff scores for each of the domains are ≥5, ≥7, and ≥7 for internalising, externalising, and attentional problems, respectively [15].

### Secondary outcomes

The following secondary outcome measures were completed by adolescents. The Patient Health Questionnaire, adolescent version (PHQ-A) assessed symptoms of depression [21]. The PHQ-A is a 9-item symptom checklist corresponding to symptoms of depression experienced in the past week and is scored on a 4-point scale (0 = *not at all*, 3 = *nearly every day*), with a score range of 0 to 36 and higher total scores reflecting more severe symptoms of depression; this scale has been validated in refugees in Jordan [22]. The Children’s Revised Impact of Events Scale (CRIES-13) assessed levels of posttraumatic stress [23]. The self-report scale consists of 13 items measuring intrusive memories, avoidance, and arousal. Items are scored on a 4-point scale (0 = *not at all*, 1 = rarely, 3 = sometimes, 5 = *often*), with a score range of 0 to 65 and higher total scores reflecting more severe symptoms of posttraumatic stress; this scale has sound psychometric properties with Middle Eastern youth [24]. Wellbeing was measured using the self-reported Warwick Edinburgh Mental Wellbeing Scale (WEMWBS), which consists of 14 items exploring the child’s thoughts and feelings in the past week [25] and has been previously used in Jordan with undergraduate students but not validated to date with Jordanian young adolescents [26]. Scores are recorded on a 5-point scale (1 = *none of the time*, 5 = *all of the time*), with a score range of 0 to 50 and higher scores indicating greater well-being. Adolescents attending school completed the Psychological Sense of School Membership (PSSM) scale to measure their sense of belonging and psychological engagement in school [27]. This self-report scale comprises 18 items that are measured on a 5-point scale (1 = *not at all true*, 5 = *completely true*), with a score range of 0 to 90 and higher scores indicating a greater sense of belonging. This scale has not been validated in an Arabic context. We assessed daily functioning with a scale developed for the EASE trial that followed methods prescribed for cross-cultural assessment ([28]; see Table C in S1 Appendix). Adolescents rated 9-items identified to be representative of their daily activities on a 4-point scale (1 = *none*, 4 = *often*), with total higher scores suggesting greater levels of impairment of daily functioning. This scale was purpose built for the EASE trial and lacks psychometric properties.

In addition, caregivers completed the caregiver version of the PSC-35 to assess their perceptions of the psychological distress of their child. This scale is identical to the child version. The Kessler Distress Scale (K6), which has been used as a measure of distress in Arabic refugees [29], was administered to measure caregivers’ level of psychological distress [30]. The K6 is a 6-item self-report scale of distress measuring symptoms experienced in the past week; each item is scored on a 5-point scale (1 = *all of the time*, 5 = *none of the time*), with a total score range of 6 to 30 and higher scores indicating more distress. Validation studies indicate that scores ≥13 indicate probable anxiety or mood disorder [31]. Parenting behaviours were assessed using the Alabama Parenting Questionnaire (APQ), which measures 5 major parenting constructs: (a) parental involvement (10 items); (b) poor supervision and monitoring (10 items); (c) positive parenting (6 items); (d) inconsistent discipline (6 items); and (e) corporal punishment (3 items) [32]. Each item is scored on a 5-point scale, with subscale score ranges from 0 to 15 to 0 to 50 and higher scores indicating greater strength of the relevant subscale. The Arabic version of this scale has robust psychometric properties [33]. To measure adolescents’ exposure to potentially traumatic events, caregivers completed a 26-item traumatic events checklist. The items were determined in collaboration with local mental health professionals to develop a trauma checklist; items were scored dichotomously.

Assessments were conducted by Arabic speaking Jordanian assessors, who received 3 days of training in research ethics, the assessment battery, general interviewing techniques, and Psychological First Aid to support them to manage any distress in participants. Assessors additionally received 2 days of practical training in conducting assessments. Assessments were conducted on portable tablets to ensure that data could be reliably collected and uploaded. As a proportion of participants were inadequately literate, assessors administered the questions and entered responses on the tablets.

### Statistical analyses

The required sample size for the trial was determined through a power calculation for individually randomised group treatment trials [34]. The estimated sample size was based on the estimated sample size required in the control arm, considering the number of EASE groups, the number of participants in each group with data at the 3-month follow-up time point, the effect size, the ratio of variance in the EASE arm versus the control arm, and the intracluster correlation coefficient [34]. In the absence of prior trials evaluating EASE, an estimate for the variance parameters was made on the basis of small datasets acquired in pilot studies in Jordan and Lebanon. This resulted in a conservative estimate of theta of 1.1 (the variance of the difference between EASE and EUC for the primary outcome) with an intraclass correlation of 0.13 (based on adolescents randomised to EASE groups). We calculated that with 20 EASE groups of 6 people each at the 3-month primary outcome time point (and assuming a 5% 2-tailed significance test and 80% power), data from 191 participants in the control arm would be needed at the 3-month follow-up to detect an effect size of 0.4 (based on the expected mean difference divided by the pooled standard deviation). This would correspond to an overall sample size of 311 at 3 months and an allocation ratio of EASE to EUC arms of 1:1.6. This ratio of allocation to EASE and EUC accommodated the effects of groups involved in EASE relative to the individually administered EUC. With a projected attrition rate of 33% loss to follow-up (based on previous scalable interventions using a 3-month follow-up [10]), we estimated the sample size required at enrolment would be approximately 470.

Baseline characteristics were compared for participants who were and were not retained at follow-up by planned comparisons with a Bonferroni adjustment to accommodate multiple comparisons (alpha = 0.002). The major analyses focused primarily on intention-to-treat. We employed linear mixed models to study differential effects of the treatment arms as this method allows the number of observations to vary between participants and thereby handles missing data by calculating estimates of trajectories using maximum likelihood estimation [35]. Fixed (intervention, time of assessment) effects and their interactions were included in the unstructured models, which provided an index of the relative effects of the treatments; time of assessment included baseline, posttreatment, and 3-month follow-up. Fixed effects parameters were tested with the Wald test (*t* test, *p* < 0.05, 2-sided) and 95% confidence intervals. Primary analyses focused on the subscales of the PSC-35 as EASE was designed with the intention to target internalising problems. The secondary outcomes (PHQ total scores, PHQ-A, CRIES-13, WEMWBS, PSSM, K6, APQ, PSC caregiver report) were analysed with the same analytic approach. Missing data was assumed to be random on the basis that participants completing the 3-month assessment and those who were missing did not differ in terms of any demographic or outcome measures at baseline. The following prespecified analyses were also conducted. We assessed the robustness of this statistical approach by conducting sensitivity analyses with only participants who completed the 3-month follow-up. To evaluate if the findings were influenced by exposure to prior traumatic experiences, analyses were repeated adjusting the models using caregivers’ baseline scores of the traumatic events checklist as a covariate. To determine the efficacy of the intervention on participants with more severe psychological disorders, we also conducted secondary analyses focusing on adolescents who scored ≥5 on the PSC internalising subscale.

To understand the potential role of EASE on parenting and adolescents’ mental health, a mediation analysis was conducted by assessing the direct and indirect effects of the intervention arm on change in the APQ subscale scores from baseline to 3-month follow-up and change in the PSC-35 subscale scores. The proposed mediation models were examined with 5,000 bootstrapped samples. These models examined the relationship between intervention arm on changes in child mental health (PSC subscale scores), with changes in parenting behaviours (APQ subscale scores) as the mediators. All analyses were overseen by an independent statistician who was blind to treatment condition.

## Results

**Fig 1:**
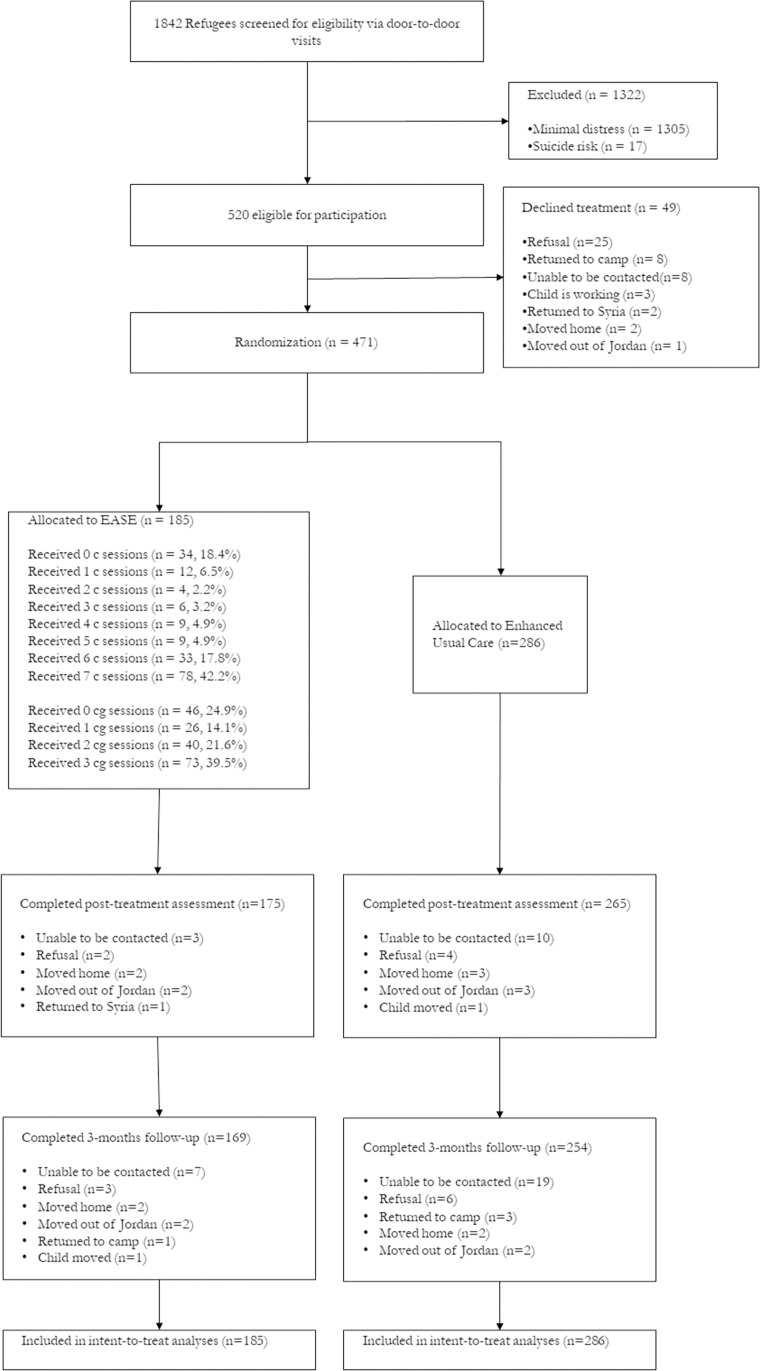
CONSORT flow diagram of progress through phases of a randomised trial comparing the EASE intervention vs. EUC in young adolescent Syrian refugees, Jordan. EASE, Early Adolescent Skills for Emotions; EUC, enhanced usual care.

Participants were enrolled between June 10, 2019 and January 11, 2020, and the final follow-up assessments were conducted on September 14, 2020. There were 520 (28.2%) adolescents meeting entry criteria, of whom 471 (90.6%) were randomised (185 into EASE and 286 into EUC). The primary outcome assessment at 3 months was conducted for 169 (91.4%) participants in EASE and 254 (88.9%) in EUC. This attrition rate (10.2%) was within the projected 33% margin on which the power analysis was calculated. Participants who were lost at follow-up did not differ from those who were retained in terms of age, gender, time since leaving Syria, trauma exposure, or baseline scores on any outcome measures (see Table D in S1 Appendix). The flowchart of participant recruitment and retention is reported in Fig 1.

Sample characteristics are presented in Table 1. Adolescents were equally distributed between genders (female; 49.5%), with a mean age 11.6 years (SD 1.3), and most participants had left their home in Syria at least 7 years ago (73.9%). The demographic factors were comparable between the 2 treatment arms. Caregivers reported that their children were exposed to an average of 6.89 (SD 3.83) traumatic events, with the most frequent being living in a war zone (60.7%), danger during flight from Syria (89.0%), seeing dead bodies (71.3%), serious injury to friends or family (67.1%), and lack of food or water (79.2%) (see Table 2).

**Table 1:** Participant characteristics.

	Total (*n* = 471)	EASE (*n* = 185)	EUC (*n* = 286)
Adolescent variables			
Female, n (%)	233 (49.5)	92 (49.7)	141 (49.3)
Age, n (%)			
10 years	135 (28.7)	52 (28.1)	83 (29.0)
11 years	80 (17.0)	28 (15.1)	52 (18.2)
12 years	131 (27.8)	57 (30.8)	74 (25.9)
13 years	72 (15.3)	29 (15.7)	43(15.0)
14 years	53 (11.3)	19 (10.3)	34 (11.9)
Education, n (%)			
No school	11 (2.3)	4 (2.2)	7 (2.4)
Primary school	330 (70.1)	128 (69.2)	202 (70.6)
Middle school	124 (26.3)	50 (27.0)	74 (25.9)
High school	6 (1.3)	3 (1.6)	3 (1.3)
Birth order, n (%)	435 (92.4)	169 (91.4)	266 (93.0)
1	167 (35.5)	60 (32.4)	107 (37.4)
2	95 (20.2)	43 (23.2)	52 (18.2)
3–4	123 (26.1)	45 (24.4)	78 (27.2)
5–6	61 (12.9)	26 (14.1)	35 (12.2)
7–10	25 (5.3)	11 (6.0)	14 (4.8)
Time since leaving Syria, n (%)			
Less than 4 years	6 (1.3)	2 (1.1)	4 (1.4)
5–6 years	117 (24.8)	45 (24.3)	72 (25.2)
7–9 years	348 (73.9)	138 (74.6)	210 (73.4)
Number of traumatic events, *M*	6.9 ± 3.8	7.1 ± 3.8	6.8 ± 3.8
Probable internalising problems, n (%)	329 (69.9)	135 (73.0)	194 (67.8)
Probable externalising problems, n (%)	295 (62.6)	111 (60.0)	184 (64.3)
Probable attentional problems, n (%)	145 (30.8)	60 (32.4)	85 (29.7)
Caregiver variables			
Female, n (%)	455 (96.6)	178 (96.2)	277 (96.9)
Age, years	38.2 ± 7.9	38.0 ± 7.3	38.4 ± 7.5
Education, n (%)			
No school	56 (11.9)	18 (9.7)	38 (13.3)
Primary school	111 (23.6)	45 (24.3)	66 (23.1)
Middle school	234 (49.7)	96 (51.9)	138 (48.3)
High school	55 (11.7)	19 (10.3)	36 (12.6)
Higher education	15 (3.2)	7 (3.8)	8 (2.8)
Probable anxiety or depression, n (%)	312 (66.4)	126 (68.1)	186 (65.3)

Continuous measures reported as means and standard deviations (±).

EASE, Early Adolescent Skills for Emotions; EUC, enhanced usual care.

**Table 2:** Participants’ reported trauma exposure.

	Total (*n* = 471)	EASE (*n* = 185)	EUC (*n* = 286)
Danger during the flight (sea, boat, border)	419 (89.0)	163 (88.1)	256 (89.5)
Lack of food or water	373 (79.2)	145 (78.4)	228 (79.7)
Another situation that was very frightening or in which your child may have felt their life was in danger	357 (75.8)	141 (76.2)	216 (75.5)
Seeing a dead body (besides at a funeral)	336 (71.3)	136 (73.5)	200 (69.9)
Serious physical injury of family member or friend	316 (67.1)	129 (69.7)	187 (65.4)
Lived in a war zone	286 (60.7)	114 (61.6)	172 (60.1)
Murder, or death due to violence, of other family member or friend	203 (43.1)	85 (45.9)	118 (41.3)
Witnessed physical assault of another person	151 (32.1)	58 (31.4)	93 (32.5)
Forced to physically harm another person	114 (24.2)	47 (25.4)	67 (286)
Exposure to toxic substance (for example, dangerous chemicals, radiation)	94 (20.0)	34 (18.4)	60 (21.0)
Serious physical injury	94 (20.0)	37 (20.0)	57 (19.9)
Ill health without access to medical care	72 (15.3)	25 (13.5)	47 (16.4)
Life-threatening illness	71 (15.1)	28 (15.1)	43 (15.0)
Witnessing killing/murder of another person	68 (14.4)	27 (14.6)	41 (14.3)
Serious accident, fire, or explosion	56 (11.9)	25 (13.5)	31 (10.8)
Refugee camp or settlement	43 (9.1)	21 (11.4)	22 (7.7)
Forced to hide	21 (4.5)	9 (4.9)	12 (4.2)
Forced separation from family member	20 (4.2)	9 (4.9)	11 (3.8)
Disappearance or kidnapping of family member or friend	20 (4.2)	10 (5.4)	10 (3.5)
Imprisonment	8 (1.7)	5 (2.7)	3 (1.0)
He/she participated in an armed conflict	7 (1.5)	1 (0.5)	6 (2.1)
Physical assault by any other person	5 (1.1)	4 (2.2)	1 (0.3)
Witness rape or sexual abuse of another person	4 (0.8)	0 (0.0)	4 (1.4)
Forced to destroy someone else’s property or possessions	3 (0.6)	0 (0.0)	3 (1.0)
Sexual assault	3 (0.6)	2 (1.1)	1 (0.3)
Having been kidnapped	1 (0.2)	1 (0.5)	0 (0.0)

EASE, Early Adolescent Skills for Emotions; EUC, enhanced usual care.

There were 329 (69.9%), 295 (62.9%), and 145 (30.8%) adolescents who met the recommended cutoffs for internalising, externalising, and attentional problems, respectively, on the PSC. There were 312 (66.4%) caregivers whose responses on the K6 indicated they had a probable anxiety or mood disorder. There were 6 severe adverse events reported during the trial (2 in EASE, 4 in EUC). These events involved suicidal risk in adolescents (2) or caregivers (2) or threat of violence to adolescents (2); none of these events were related to the interventions.

The mean number of EASE sessions attended by adolescents was 4.67 (SD 2.79), with 120 (64.9%) participants attending at least 5 sessions. Caregivers attended on average 1.76 (SD 1.22) sessions, with 113 (61.1%) participants attending at least 2 sessions; nearly all caregivers attending sessions were female (183, 98.9%). In terms of fidelity, 38 EASE sessions were observed and rated by supervisors; 96% of sessions were rated as completing the prescribed protocol. The mean competency rating was 2.89 (possible range 0 to 3). Assessors correctly guessed the participant’s allocation condition at chance rates at both the posttreatment (49.0%) and follow-up (56.5%) assessments. No participants disclosed their allocation during the posttreatment assessment, and one participant in EASE disclosed their allocation at the follow-up assessment.

The primary and secondary outcomes at each time point are presented in Table 3. At the 3-month follow-up assessment, participants who received EASE had greater reductions in PSC-internalising scores than those receiving EUC (estimated mean difference 0.69, 95% CI 0.19 to 1.19; *p* = 0.007; effect size, 0.38). There were no differences in terms of externalising (estimated mean difference 00.24, 95% CI −0.53 to 0.52; *p* = 0.97; effect size, −0.10), attentional (estimated mean difference −0.01, 95% CI −0.51 to 0.54; *p* = 0.97; effect size, −0.01), or PSC total (estimated mean difference 0.04, 95% CI −2.66 to 2.74; *p* = 0.97; effect size, 0.00) scores. There were also no significant differences between conditions on other secondary outcome measures for adolescents.

**Table 3:** Summary statistics and results from mixed model analysis of primary and secondary outcomes.

		Descriptive statistics		Mixed model analysis		
	Visit	Intervention (*n* = 185)	EUC (*n* = 286)	Difference in LS mean (95% CI)	*P*-value	Effect size^a^
		Estimated mean (SE)	Estimated mean (SE)			
Child reported outcomes						
Primary outcomes						
PSC internalising	Baseline	5.57 (0.14)	5.29 (0.11)			
	9-week	2.90 (0.14)	3.33 (0.11)	0.71 (0.22, 1.20)	0.004	0.39
	3 months	2.96 (0.15)	3.38 (0.12)	0.69 (0.19, 1.19)	0.007	0.38
PSC externalising	Baseline	7.24 (0.19)	7.34 (0.15)			
	9-week	4.36 (0.18)	4.57 (0.14)	0.10 (−0.55, 0.75)	0.75	0.04
	3 months	4.54 (0.18)	4.40 (0.14)	0.24 (−0.43, 0.91)	0.49	0.10
PSC attention	Baseline	5.56 (0.14)	5.55 (0.12)			
	9-week	2.96 (0.14)	3.31 (0.11)	0.36 (−0.14, 0.86)	0.16	0.19
	3 months	3.23 (0.16)	3.21(0.13)	−0.01 (−0.51, 0.54)	0.97	−0.01
PSC total	Baseline	32.59 (0.64)	32.48 (0.52)			
	9-week	18.42 (0.80)	20.16 (0.65)	1.86 (−0.48, 4.20)	0.12	0.21
	3 months	18.90 (0.84)	18.83 (0.69)	0.04 (−2.66, 2.74)	0.97	0.00
Secondary outcomes						
CRIES	Baseline	24.19 (0.89)	23.29 (0.71)			
	9-week	18.39 (0.63)	18.23 (0.50)	0.74 (−1.93, 3.40)	0.59	0.06
	3 months	18.79 (0.72)	18.87 (0.58)	0.98 (−1.86, 3.85)	0.50	0.08
PHQ-A	Baseline	15.06 (0.43)	15.39 (0.27)			
	9-week	12.80 (0.36)	12.37 (0.29)	−0.75 (−2.17, 0.67)	0.30	−0.13
	3 months	12.42 (0.35)	12.36 (0.28)	−0.37 (−1.86, 1.11)	0.62	−0.06
Functioning	Baseline	16.71 (0.53)	16.76 (0.43)			
	9-week	13.27 (0.48)	13.68 (0.38)	0.36 (−1.45, 2.17)	0.69	0.05
	3 months	14.52 (0.53)	14.44 (0.42)	−0.13 (−2.18, 1.93)	0.90	−0.02
WEBWBS	Baseline	40.72 (0.69)	39.62 (0.56)			
	9-week	45.08 (0.54)	44.96 (0.44)	0.98 (−1.20, 3.16)	0.38	0.10
	3 months	44.87 (0.60)	45.11 (0.49)	1.35 (−0.97, 3.66)	0.25	0.14
School	Baseline	2.79 (0.05)	2.86 (0.04)			
	9-week	3.13 (0.04)	3.17 (0.03)	0.04 (−0.19, 0.12)	0.66	0.07
	3 months	2.98 (0.04)	2.99 (0.03)	−0.07 (−0.23, 0.10)	0.42	−0.12
Caregiver reported outcomes						
Alabama involvement	Baseline	29.58 (0.45)	29.88 (0.36)			
	9-week	30.58 (0.43)	30.71 (0.35)	−0.16 (−1.64, 1.31)	0.83	−0.03
	3 months	30.68 (0.42)	30.50 (0.35)	−0.48 (−1.98, 1.01)	0.53	−0.08
Alabama supervision	Baseline	17.35 (0.47)	17.46 (0.38)			
	9-week	17.38 (0.45)	16.46 (0.37)	−1.03 (−2.59, 0.53)	0.19	−0.16
	3 months	16.78 (0.43)	15.74 (0.35)	1.16 (−2.85, 0.53)	0.18	0.32
Alabama positive parenting	Baseline	19.27 (0.27)	19.70 (0.22)			
	9-week	29.82 (0.27)	19.95 (0.22)	0.19 (0.72, 1.10)	0.68	0.05
	3 months	19.97 (0.27)	19.95 (0.22)	−0.03 (−0.9, 1.00)	0.99	−0.01
Alabama discipline	Baseline	15.25(0.27)	15.23 (0.22)			
	9-week	13.95 (0.27)	13.91 (0.22)	0.53 (−0.38, 1.46)	0.25	0.00
	3 months	14.14 (0.28)	13.94 (0.23)	1.23 (0.26, 2.19)	0.01	0.34
Alabama punishment	Baseline	6.64 (0.18)	6.59 (0.14)			
	9-week	6.15 (0.15)	5.69 (0.13)	−0.40 (−0.96, 0.15)	0.15	−0.17
	3 months	5.74 (0.15)	5.75 (0.12)	0.05 (−0.52, 0.63)	0.85	0.02
PSC internalising—caregiver	Baseline	4.40 (0.18)	4.53 (0.14)			
	9-week	2.79 (0.17)	3.02 (0.14)	−0.10 (−0.48, 0.67)	0.74	−0.04
	3 months	2.69 (0.17)	2.68 (0.14)	0.14 (−0.23, 0.10)	0.68	0.06
PSC externalising—caregiver	Baseline	5.44 (0.20)	4.94 (0.16)			
	9-week	3.47 (0.18)	3.87 (0.14)	0.91 (0.26, 1.56)	0.006	0.34
	3 months	3.78 (0.19)	3.73 (0.16)	0.46 (−0.27, 1.20)	0.22	0.17
PSC attention—caregiver	Baseline	5.58 (0.17)	5.53 (0.14)			
	9-week	3.75 (0.16)	3.75 (0.16)	0.31 (−0.27, 0.88)	0.29	0.13
	3 months	3.90 (0.16)	3.94 (0.13)	0.10 (−0.52, 0.70)	0.77	0.21
PSC total—caregiver	Baseline	27.00 (0.70)	27.93 (0.87)			
	9-week	20.82 (0.69)	19.58 (0.84)	−2.17 (−4.86, 0.51)	0.11	−0.19
	3 months	19.18 (0.71)	19.31 (0.86)	−0.82 (−4.14, 2.51)	0.63	−0.07
K6	Baseline	15.44 (0.38)	14.47 (0.31)			
	9-week	16.89 (0.34)	16.86 (0.28)	0.94 (0.31, 2.19)	0.14	0.18
	3 months	16.74 (0.35)	17.72 (0.28)	1.95 (0.71, 3.19)	0.002	0.38

^a^Effect size was calculated by the difference in LS means between intervention and EUC from mixed model divided by the pooled standard deviation at each visit. Number of adolescents completing baseline, 9-week, and 3-month assessments were 471, 440, and 423, respectively. Number of caregivers completing baseline, 9-week, and 3-month assessments were 470, 440, and 422, respectively.

CRIES, Children’s Revised Impact of Events Scale; EASE, Early Adolescent Skills for Emotions; EUC, enhanced usual care; K6, Kessler Psychological Distress Scale Version (total score range: 6–30; higher scores indicate more severe psychological distress); LS, least square; PHQ-9A, Patient Health Questionnaire Adolescent Version (total score range: 0–27; higher scores indicate more severe depression); PSC, paediatric symptom checklist; WEBWBS, Warwick Edinburgh Mental Wellbeing Scale.

Relative to EUC, caregivers in the EASE condition had greater reductions in psychological distress (estimated mean difference 1.95, 95% CI 0.71 to 3.19; *p* = 0.002, effect size, 0.38) and inconsistent disciplinary parenting (estimated mean difference 1.23, 95% CI 0.26 to 2.19; *p* = 0.01, effect size, 0.34) at follow-up. No differences were observed for parental involvement, positive parenting, poor supervision and monitoring, or corporal punishment.

The analysis that focused on adolescents who met the criterion for internalising problems on the PSC at baseline indicated EASE resulted in greater reductions in internalising symptoms than EUC (estimated mean difference 0.93, 95% CI 0.41 to 1.45; *p* < 0.001; effect size, 0.52) and caregiver’s inconsistent disciplinary parenting (estimated mean difference 1.55, 95% CI 0.42 to 2.69; *p* = 0.008; effect size, 0.43) (see Table 4). This subgroup also indicated that EASE led to apparent reductions in psychological distress relative to EUC (estimated mean difference 1.43, 95% CI −0.07 to 2.93; *p* = 0.06; effect size, 0.28) although this did not reach statistical significance.

**Table 4:** Summary statistics and results from mixed model analysis of primary and secondary outcomes for adolescents with internalising problems.

		Descriptive statistics		Mixed model analysis		
Primary and secondary outcomes	Visit	Intervention	EUC	Difference in LS mean (95% CI)	*P*-value	Effect size^a^
		Estimated mean (SE)	Estimated mean (SE)			
Child reported outcomes						
Primary outcomes						
PSC internalising	Baseline	6.52 (0.11)	6.25 (0.09)			
	9-week	2.98 (0.17)	3.33 (0.14)	0.62 (0.12, 1.13)	0.016	0.34
	3 months	3.52 (0.15)	3.51 (0.15)	0.93 (0.41, 1.45)	<0.001	0.52
PSC externalising	Baseline	7.62 (0.22)	7.58 (0.18)			
	9-week	4.42 (0.20)	4.56 (0.17)	0.18 (−0.59, 0.96)	0.64	0.07
	3 months	4.58 (0.21)	4.51 (0.18)	−0.02 (−0.80, 0.76)	0.97	−0.01
PSC attention	Baseline	6.50 (0.11)	6.44 (0.10)			
	9-week	3.03 (0.16)	3.32 (0.14)	0.35 (−0.16, 0.86)	0.38	0.18
	3 months	3.16 (0.19)	3.26(0.16)	0.17 (−0.38, 0.71)	0.55	0.09
PSC total	Baseline	33.33 (0.62)	33.39 (0.62)			
	9-week	18.34 (0.95)	19.65 (0.79)	1.26 (−1.53, 4.05)	0.38	0.21
	3 months	18.38 (1.01)	19.12 (0.85)	0.69 (−2.64, 4.02)	0.68	0.00
Secondary outcomes						
CRIES	Baseline	24.66 (1.05)	24.30 (0.87)			
	9-week	18.83 (0.76)	18.17 (0.63)	−0.30 (−3.54, 2.93)	0.86	−0.03
	3 months	18.22 (0.82)	18.81 (0.69)	0.95 (−2.40, 4.30)	0.58	0.08
PHQ-A	Baseline	15.32 (0.51)	15.50 (0.43)			
	9-week	12.75 (0.43)	12.40 (0.36)	−0.54 (−2.25, 1.18)	0.54	−0.09
	3 months	12.18 (0.42)	12.73 (0.35)	0.37 (−1.48, 2.18)	0.69	0.06
Functioning	Baseline	17.30 (0.62)	17.12 (0.52)			
	9-week	13.20 (0.56)	13.26 (0.46)	0.24 (−1.91, 2.40)	0.83	0.03
	3 months	14.25 (0.50)	14.50 (0.50)	0.43 (−1.99, 2.84)	0.73	0.06
WEBWBS	Baseline	41.9 (0.82)	39.21 (0.68)			
	9-week	44.53 (0.63)	45.09 (0.52)	2.44 (−0.18, 5.06)	0.07	0.26
	3 months	45.11 (0.73)	45.19 (0.61)	1.96 (−0.83, 4.76)	0.17	0.21
School	Baseline	2.80 (0.06)	2.92 (0.05)			
	9-week	3.07 (0.05)	3.17 (0.04)	−0.03 (−0.21, 0.15)	0.74	−0.05
	3 months	2.98 (0.05)	3.00 (0.04)	−0.10 (−0.29, 0.09)	0.32	−0.17
Caregiver reported outcomes						
Alabama involvement	Baseline	29.50 (0.52)	29.94 (0.44)			
	9-week	30.11 (0.49)	30.55 (0.41)	−0.01 (−1.79, 1.78)	0.99	0.00
	3 months	30.49 (0.51)	30.41 (0.43)	−0.52 (−2.27, 1.23)	0.60	−0.08
Alabama supervision	Baseline	17.60 (0.56)	17.58 (0.46)			
	9-week	17.60 (0.55)	16.38 (0.46)	−1.19 (−3.06, 0.68)	0.21	−0.19
	3 months	17.06 (0.52)	15.69 (0.43)	−1.34 (−0.38, 0.69)	0.20	−0.37
Alabama positive parenting	Baseline	19.67 (0.31)	19.78 (0.26)			
	9-week	19.61 (0.32)	19.82 (0.26)	0.08 (−1.10, 1.16)	0.88	0.02
	3 months	19.84 (0.32)	20.03 (0.27)	0.07 (−1.07, 1.20)	0.91	0.03
Alabama discipline	Baseline	15.73 (0.32)	15.28 (0.27)			
	9-week	13.50 (0.27)	13.72 (0.27)	0.67 (−0.44, 1.78)	0.24	0.19
	3 months	13.21 (0.32)	14.31 (0.27)	1.55 (0.42, 2.69)	0.008	0.43
Alabama punishment	Baseline	6.59 (0.21)	6.58 (0.18)			
	9-week	6.08 (0.18)	5.74 (0.15)	−0.32 (−0.99, 0.35)	0.35	−0.13
	3 months	5.77 (0.18)	5.89 (0.15)	0.12 (−0.60, 0.84)	0.74	0.05
PSC internalising—caregiver	Baseline	4.44 (0.21)	4.62 (0.17)			
	9-week	2.78 (0.21)	3.00 (0.17)	0.04 (−0.66, 0.75)	0.91	0.02
	3 months	2.69 (0.21)	2.70 (0.18)	−0.16 (−0.97, 0.65)	0.70	−0.07
PSC Externalising—caregiver	Baseline	5.61 (0.24)	4.96 (0.20)			
	9-week	3.40 (0.21)	3.82 (0.17)	1.07 (0.30, 1.84)	0.007	0.40
	3 months	3.72 (0.23)	3.72 (0.19)	0.65 (−0.24, 1.53)	0.15	0.24
PSC attention—caregiver	Baseline	5.56 (0.17)	5.56 (0.17)			
	9-week	3.77 (0.19)	3.96 (0.16)	0.08 (−0.61, 0.78)	0.82	0.03
	3 months	3.91 (0.19)	3.90 (0.16)	−0.12 (−0.86, 0.61)	0.75	−0.05
PSC total—caregiver	Baseline	27.06 (0.88)	27.86 (1.06)			
	9-week	20.62 (0.85)	19.21 (1.01)	−2.20 (−0.52, 1.12)	0.19	−0.19
	3 months	19.38 (0.86)	18.97 (1.03)	−1.20 (−5.29, 2.87)	0.56	−0.07
K6	Baseline	15.52 (0.46)	14.16 (0.38)			
	9-week	16.62 (0.40)	16.52 (0.34)	1.25 (−0.22, 2.72)	0.09	0.24
	3 months	17.12 (0.38)	17.70 (0.32)	1.43 (−0.07, 2.93)	0.06	0.28

^a^Effect size was calculated by the difference in LS means between intervention and EUC from mixed model divided by the pooled standard deviation at each visit. Number of adolescents completing baseline, 9-week, and 3-month assessments were 329, 301, and 288, respectively. Number of caregivers completing baseline, 9-week, and 3-month assessments were 328, 307, and 292, respectively.

CRIES, Children’s Revised Impact of Events Scale; EASE, Early Adolescent Skills for Emotions; EUC, enhanced usual care; K6, Kessler Psychological Distress Scale Version (total score range: 6–30; higher scores indicate more severe psychological distress); LS, least square; PHQ-9A, Patient Health Questionnaire Adolescent Version (total score range: 0–27; higher scores indicate more severe depression); PSC, paediatric symptom checklist; WEBWBS, Warwick Edinburgh Mental Wellbeing Scale; presence of internalising problems defined as paediatric symptom checklist—internalising subscale score ≥5.

The sensitivity analysis that focused only on participants that were retained at the 3-month follow-up did not change the pattern of results observed in the linear mixed models analyses (see Table E in S1 Appendix). Results of the covariate-adjusted analysis that controlled for trauma exposure were consistent with those in the primary analysis; this indicated that degree of trauma exposure did not impact results insofar as those in the EASE condition had greater reductions in internalising problems, caregiver distress, and inconsistent disciplinary parenting relative to EUC (see Table F in S1 Appendix).

**Fig 2:**
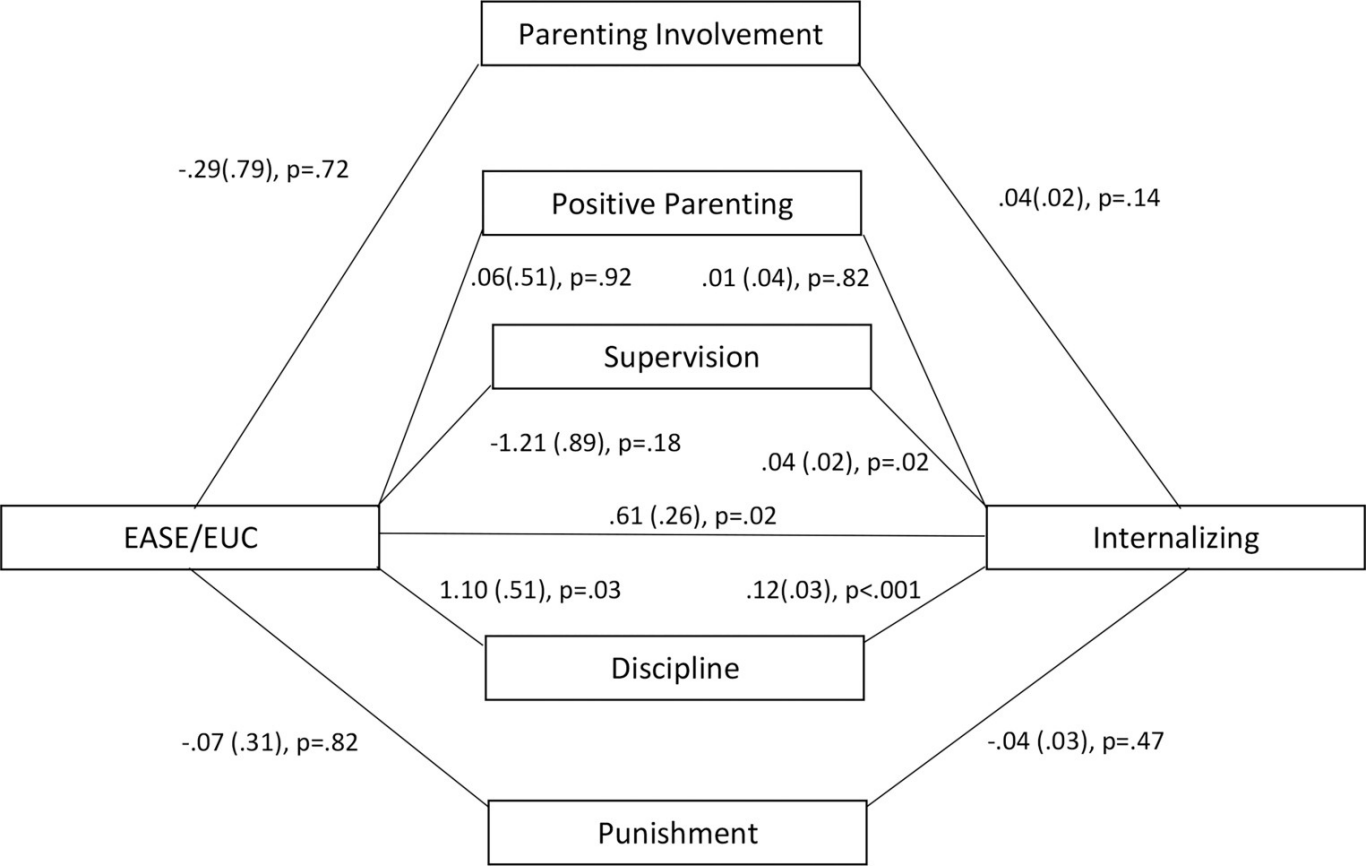
Path model of relationship between intervention, change in parenting style, and change in children’s mental health. Path model demonstrates that for refugees in gPM+, the more disciplinary parenting decreased (as measured by the Alabama Parenting Questionnaire) there was a greater decrease in their children’s and internalising problems (as measured by the Pediatric Symptom Checklist). Values are unstandardized coefficients (standard error). EASE, Early Adolescent Skills for Emotions; EUC, enhanced usual care.

The mediation analysis indicated an indirect path between receiving EASE and greater reductions in the adolescents’ reported internalising symptoms via reductions in caregivers’ inconsistent disciplinary behaviours (β = 0.13, SE 0.07; 95% CI 0.01, 0.28). These patterns suggest that EASE was associated with improvements in adolescents’ internalising problems when there was a decrease in inconsistent disciplinary parenting (see Fig 2). The mediation analyses using PSC externalizing, attentional, and total scores were not significant.

## Discussion

The primary finding of this trial was that the brief, group-based EASE intervention resulted in a greater reduction of internalising problems in young adolescent refugees than usual care. There were also benefits for the caregivers in that those in the EASE arm reported greater reductions in psychological distress and inconsistent disciplinary parenting relative to usual care. Finally, exploratory analysis indicated that the reduction of inconsistent parenting behaviour in caregivers who received EASE may have led to reductions in internalising problems in their children. It is important to note that EASE did not impact on the PSC total scores, or externalising or attentional problems, or other secondary outcomes.

The observation that EASE achieved a moderate effect relative to the comparison arm is consistent with most adult scalable nonspecialist interventions [10,11]. The benefit to participants in reducing internalising symptoms is consistent with the explicit goal of EASE to address internalising symptoms by focusing on skills in stress management, behaviour activation, and problem management [12]. The capacity of EASE to reduce internalising symptoms is an important finding because of the strong evidence that refugee adolescents experience elevated rates of anxiety and depression [1], and there is currently a lack of evidence-based effective, scalable interventions to address these problems [3].

Despite the capacity of EASE to reduce internalising problems in young adolescents relative to EUC, 21.9% of these adolescents still met the cutoff for internalising problems at 3-month follow-up. Moreover, EASE did not have apparent benefits in mitigating externalising behaviour, attentional problems, overall PSC severity, PTSD, or functioning. This may not be surprising because the EASE intervention was intentionally developed to address internalising problems. The current findings point to the need for development of scalable interventions that can address externalising and other psychological problems in young adolescents in LMICs. The need remains to evaluate scalable programs that can effectively target externalising problems, and this issue needs to be addressed as part of a comprehensive mental health strategy for young adolescents in LMICs.

It should be noted that nearly half of the follow-up assessments for this trial occurred when the sites in Amman were severely affected by lockdowns and unemployment caused by the Coronavirus Disease 2019 (COVID-19) pandemic. The problems caused by this situation may have driven the increased distress in caregivers in both arms, as reflected by higher K6 scores at the 3-month assessment. The observation that the caregivers who took part in the EASE intervention had less distress than those in EUC suggests that the EASE program served as a relative buffer against the increased stressors occurring in Jordan during the pandemic.

The mediation analysis found that in the case of participants in the EASE condition, as inconsistent disciplinary parenting behaviour reduced from baseline to follow-up, there was a decrease in adolescents’ internalising problems across the same time frame. Harsh disciplinary parenting (including verbal or physical threats or acts) is associated with emotional and behavioural problems in adolescents [36]. The mediation analysis indicates that the content of the caregiver sessions may have impacted on the reduction of disciplinary parenting in ways that reduced emotional difficulties in their children. Recent research with parenting programs of Syrian refugees have found that caregiver support interventions have potential to positively impact children’s psychological wellbeing [37]. Taken together, this evidence points to the possibility that focusing on parenting practices may have psychological benefits for refugee and other adolescents in LMICs.

Strengths of this study included random allocation, excellent retention at follow-up assessment with 91.4% of participants in EASE and 88.9% in EUC assessed at 3-months, adherence to the treatment protocols, verified blind assessments, use of nonspecialist providers, and extensive cultural adaptation of EASE for Syrian refugees. Limitations included reliance on some measures (including the Psychological Sense of School Membership and the functioning measure) that have not been culturally validated in Arabic adolescents, the treatment arms were not matched for weekly contact, and the majority of caregivers were female (typically mothers). Future studies should aim to address this because of the potential for male caregivers to express harsh disciplinary parenting.

In the context of the dearth of scalable interventions for young adolescents who have emotional difficulties in LMICs, the current findings provide initial evidence that EASE can be an effective program to mitigate internalising problems in young adolescents. Although this trial was conducted with young adolescent refugees, the broader intention of EASE to mitigate internalising problems in any young adolescents in LIMCs where there are insufficient mental health resources and task-sharing approaches are more appropriate. It should be noted that approximately 40% of adolescents attended 4 or fewer sessions of the scheduled 7 sessions of EASE, which raises questions about alternate approaches to ensure that adequate dosage of EASE is achieved. It is possible that future iterations of EASE could be tried to achieve better group attendance, such as overcoming transportation barriers, competition with the priority to attend employment opportunities, or using digital technology to conduct group sessions.

The rationale behind the EASE intervention is to develop an efficacious program that can be scaled up in countries with insufficient mental health infrastructure and few mental health specialists to enable access to young adolescents with mental health needs. The EASE program is scalable insofar as it can be delivered by lay providers who trained for only 8 days and is a relatively brief intervention. It should be acknowledged that there remains a need to better understand the cost effectiveness of EASE because the program requires 16.5 hours of facilitators’ labour per group, in addition to the 8 days of facilitator training. Full cost benefit analysis is needed to determine if the labour required for this program justifies the benefits relative to possible more affordable programs. After proper cost effectiveness studies are completed, the utility of EASE will need to be determined in countries where there are no other viable mental health programs available.

The capability of this intervention to be delivered in group format and by nonspecialist providers after brief training points to the potential for the EASE program to be a sustainable mental health program in settings with limited health resources. The finding that EASE did not impact externalising or functioning points to the need for further development of effective strategies to address these issues.

## Supporting information

S1 CONSORT Checklist CONSORT, Consolidated Standards of Reporting Trials. https://doi.org/10.1371/journal.pmed.1003862.s002.
(PDF)

S1 Appendix Supplementary materials and results. Table A. Selection criteria. Table B. Outline of EASE program. Table C. Functioning measure. Table D. Baseline participant characteristics of participants in EASE and enhanced usual care. Table E. Mixed model analysis of primary and secondary outcomes of participants who completed 3-month assessment. Table F. Sensitivity analyses controlling for trauma exposure.
(DOCX)

S1 Text Trial Protocol. (DOCX)

S1 File World Health Organization ethics approval. (PDF)

S2 File Local Jordan approval. (PDF)

## Abbreviations

APQ: Alabama Parenting Questionnaire
COVID-19: Coronavirus Disease 2019
CRIES: Children’s Revised Impact of Events Scale
EASE: Early Adolescent Skills for Emotions
EUC: enhanced usual care
IFH: Institute for Family Health
LMIC: low- and middle-income country
PHQ-A: Patient Health Questionnaire, adolescent version
PSC: Paediatric Symptom Checklist
PSSM: Psychological Sense of School Membership
PTSD: posttraumatic stress disorder
RCT: randomised controlled trial
WEMWBS: Warwick Edinburgh Mental Wellbeing Scale
WHO: World Health Organization
