# Supplementary material for: Effectiveness of a brief group behavioural intervention on psychological distress in young adolescent Syrian refugees: A randomised controlled trial
Source: PLoS Med. 2022 Aug 12;19(8):e1004046. doi: 10.1371/journal.pmed.1004046 (PMC9374250; doi:10.1371/journal.pmed.1004046)
Supplement: S1 CONSORT Checklist — https://doi.org/10.1371/journal.pmed.1003862.s002. (PDF) [file pmed.1004046.s001.pdf]

Print Form

Table. CONSORT 2010 Checklist of Information to Include When Reporting a Randomized Trial[a]

Section and Topic	Item No.	Checklist Item	Reported on Page No. P = Paragraph
**Title and abstract**			
	1a	Identification as a randomized trial in the title	Title
	1b	Structured summary of trial design, methods, results, and conclusions (for specific guidance see CONSORT for abstracts) P = Paragaph	Abstract
**Introduction**			
Background and objectives	2a	Scientific background and explanation of rationale	Intro:P1-
	2b	Specific objectives or hypotheses	Intro: P4
**Methods**			
Trial design	3a	Description of trial design (such as parallel, factorial) including allocation ratio	Method:
	3b	Important changes to methods after trial commencement (such as eligibility criteria), with reasons	NA
Participants	4a	Eligibility criteria for participants	Method:
	4b	Settings and locations where the data were collected	Method:
Interventions	5	The interventions for each group with sufficient details to allow replication, including how and when they were actually administered	Method:
Outcomes	6a	Completely defined prespecified primary and secondary outcome measures, including how and when they were assessed	Method:
	6b	Any changes to trial outcomes after the trial commenced, with reasons	NA
Sample size	7a	How sample size was determined	Method:
	7b	When applicable, explanation of any interim analyses and stopping guidelines	NA
Randomization			
Sequence generation	8a	Method used to generate the random allocation sequence	Method:
	8b	Type of randomization; details of any restriction (such as blocking and block size)	Method:
Allocation concealment mechanism	9	Mechanism used to implement the random allocation sequence (such as sequentially numbered containers), describing any steps taken to conceal the sequence until interventions were assigned	Method:
Implementation	10	Who generated the random allocation sequence, who enrolled participants, and who assigned participants to interventions	Method:
Blinding	11a	If done, who was blinded after assignment to interventions (for example, participants, care providers, those assessing outcomes) and how	Method:
	11b	If relevant, description of the similarity of interventions	NA
Statistical methods	12a	Statistical methods used to compare groups for primary and secondary outcomes	Method:
	12b	Methods for additional analyses, such as subgroup analyses and adjusted analyses	Method:
**Results**			
Participant flow (a diagram is strongly recommended)	13a	For each group, the numbers of participants who were randomly assigned, received intended treatment, and were analyzed for the primary outcome	Fig 1
	13b	For each group, losses and exclusions after randomization, together with reasons	Fig 1
Recruitment	14a	Dates defining the periods of recruitment and follow-up	Results:
	14b	Why the trial ended or was stopped	NA
Baseline data	15	A table showing baseline demographic and clinical characteristics for each group	Table 1
Numbers analyzed	16	For each group, number of participants (denominator) included in each analysis and whether the analysis was by original assigned groups	Fig 1
Outcomes and estimation	17a	For each primary and secondary outcome, results for each group, and the estimated effect size and its precision (such as 95% confidence interval)	Table 3
	17b	For binary outcomes, presentation of both absolute and relative effect sizes is recommended	NA
Ancillary analyses	18	Results of any other analyses performed, including subgroup analyses and adjusted analyses, distinguishing prespecified from exploratory	esults: F
Harms	19	All important harms or unintended effects in each group (for specific guidance see CONSORT for harms)	esults: F
**Comment**			
Limitations	20	Trial limitations, addressing sources of potential bias, imprecision, and, if relevant, multiplicity of analyses	Discussi
Generalizability	21	Generalizability (external validity, applicability) of the trial findings	Discussi
Interpretation	22	Interpretation consistent with results, balancing benefits and harms, and considering other relevant evidence	Discussi
**Other information**			
Registration	23	Registration number and name of trial registry	Abstract
Protocol	24	Where the full trial protocol can be accessed, if available	Suppl
Funding	25	Sources of funding and other support (such as supply of drugs), role of funders	Financia

[a] We strongly recommend reading this statement in conjunction with the CONSORT 2010 Explanation and Elaboration for important clarifications on all the items. If relevant, we also recommend reading CONSORT extensions for cluster randomized trials, noninferiority and equivalence trials, nonpharmacological treatments, herbal interventions, and pragmatic trials. Additional extensions are forthcoming: for those and for up-to-date references relevant to this checklist, see http://www.consort-statement.org.

©2010 American Medical Association. All rights reserved.
