# Supplementary material for: Effectiveness of a brief group behavioural intervention on psychological distress in young adolescent Syrian refugees: A randomised controlled trial
Source: PLoS Med. 2022 Aug 12;19(8):e1004046. doi: 10.1371/journal.pmed.1004046 (PMC9374250; doi:10.1371/journal.pmed.1004046)
Supplement: S1 Appendix — Table A. Selection criteria. Table B. Outline of EASE program. Table C. Functioning measure. Table D. Baseline participant characteristics of participants in EASE and enhanced usual care. Table E. Mixed model analysis of primary and secondary outcomes of participants who completed 3-month assessment. Table F. Sensitivity analyses controlling for trauma exposure. (DOCX) [file pmed.1004046.s002.docx]

Supplementary Appendix

This appendix has been provided by the authors to give readers additional information about their work.

**Table of Contents**

**Supplementary Tables**

Table A. Selection Criteria …………………………………………………………. 3 Table B. Outline of EASE Program ………………………………………………… 4-5

Table C: Functioning Measure ……………………………………………………… 6

Table D. Baseline participant characteristics of participants in EASE

and Enhanced Usual Care …………………………………………………….. 7

Table E: Mixed model analysis of primary and secondary outcomes of participants who

completed three-month assessment………………………………………… 8-10

Table F: Sensitivity analyses controlling for trauma exposure ……………………… 11-13

Table A. Selection Criteria

| **Inclusion Criteria** |
| --- |
| Participants were enlisted in the trial if they met the following inclusion criteria when assessed at home visits during the screening process in Amman:   1. Syrian refugee; 2. Aged 10-14 years; 3. Resided with a related caregiver who could provide legal consent; 4. Scores ≥15 on the Paediatric Symptom Scale (PSC-17)   Only one child, selected by the caregiver, was recruited per household in order to minimize the burden on the family and reduce likelihood of contamination of the intervention between participants. |
| **Exclusion Criteria** |
| 1. Unaccompanied minor; 2. Minors with an unrelated caregiver who could provide legal consent; 3. Significant developmental, cognitive, or neurological impairments as determined by four items from an adapted version of the Ten Questions instrument; 4. Imminent risk of suicide; 5. Imminent risk of child abuse and requiring urgent child protection   Any potential participants who met the exclusion criteria were referred to specialized services within IFH or another organization according to Inter-Agency Standard Operating Procedures. |

Table B. Outline of EASE Program

| **Session** | **Content** |
| --- | --- |
| **Adolescent Sessions** | |
| 1: Understanding my Feelings | - Welcome and group guidelines - Introduction to the storybook - Psychoeducation: understanding my feelings and identifying personal feelings |
| 2: Calming my Body | - Review home practice - Education about feelings and my body - Calming my body strategy (slow breathing) |
| 3: Changing my Actions 1 | - Review home practice - Education about feelings and actions - Changing my actions strategy |
| 4: Changing my Actions 2 | - Review home practice - Continue changing my actions strategy |
| 5: Managing my Problems 1 | - Review home practice - Understanding common problems (in young adolescents) - Managing my problems strategy (problem solving) |
| 6: Managing my Problems 2 | - Review home practice - Continue managing my problems strategy |
| 7: Brighter Futures | - Review home practice - Education about relapse prevention - Closing ceremony/activity |
| **Caregiver Sessions** | |
| 1: Understanding Sadness, Worry and Stress | - Welcome and group guidelines - Appreciating caregiver strengths - Psychoeducation: common signs of sadness, worry and stress in young adolescents - Review EASE youth sessions - Responding to a child’s feelings through active listening and slow breathing strategy - Quality time with children |
| 2: Boosting Confidence | - Review home practice - Boosting confidence in young adolescents - Appreciating children’s strengths - Praise - Alternatives to physical punishment |
| 3: Caregiver Self-care and Brighter Futures | - Review home practice - Caregiver challenges and self-care - Review caregiver sessions - Education about relapse prevention |

Table C. Functioning Impairment Scale

| **Functioning Impairment Scale** | |
| --- | --- |
|  | 1                   2                 3                 4  None*         A little     Moderate     Often  *also if N/a |
| **[DF1]** In the past week, have you had any difficulty with helping your caregivers and other family members *(e.g. doing household  chores, taking care of siblings)* | 1                  2                  3                 4 |
| **[DF2]** In the past week, have you had any difficulty with showing respect and affection to adults that are important to you *(e.g. being polite to the parents, listen to their advice, listen to their request, kiss goodbye when you are leaving)* | 1                  2                  3                 4 |
| **[DF3]** In the past week, have you had any difficulty with spending nice/pleasurable time with your siblings and any other family members? | 1                  2                  3                 4 |
| **[DF4]**  In the past week, have you had any difficulty with spending time on praying or other religious activities *(e.g. reading the Quran/bible, going to the mosque/church)* | 1                  2                  3                 4 |
| **[DF5]** In the past week, have you had any difficulty with eating and enjoying food? | 1                  2                  3                 4 |
| **[DF6]** In the past week, have you had any difficulty with taking care of your hygiene *(e.g. washing yourself, washing your clothes, personal grooming)* | 1                  2                  3                 4 |
| **[DF7]** In the past week, have you had any difficulty with playing with your friends *(e.g. meet and hang out, playing games, making music, football (for boys), racing, as well as dance and clap* | 1                  2                  3                 4 |
| **[DF8]** In the past week, have you had any difficulty with entertaining yourself? *(e.g. playing with video games, doing arts and crafts, singing and dancing and individual sports)* | 1                  2                  3                 4 |
| **[DF9]** In the past week, have you had any difficulty with studying or doing well in school? *(e.g. doing homework, working hard in school, good participation in class)* | 1                  2                  3                 4 |
| **[DF10]***Another activity* that you yourself can think of that is important to you:  __________________________________________ | 1                  2                  3                 4 |

Table D. Baseline participant characteristics of participants in EASE and Enhanced Usual Care

|  | EASE  (n = 185) | EUC  (n = 286) |
| --- | --- | --- |
| Female, n (%) | 92 (49.7) | 141 (49.3) |
| Child Age, y (SD) | 11.6 (1.3) | 11.6 (1.4) |
| Time Since Leaving Syria, y (SD) | 2.74 (0.47) | 2.72 (0.48) |
| Trauma exposure total (SD) | 7.07 (3.84) | 6.76 (3.82) |
| Internalising problems, m (SD) | 5.57 (2.00) | 5.29 (1.78) |
| Externalising problems, m (SD) | 5.44 (2.72) | 4.93 (2.68) |
| Attentional problems, m (SD) | 5.56 (1.99) | 5.55 (1.91) |
| Posttraumatic stress, m (SD) | 24.19 (12.06) | 20.29 (12.00) |
| Depression, m (SD) | 15.06 (5.82) | 15.38 (5.95) |
| Functioning, m (SD) | 16.71 (6.96) | 16.76 (7.44) |
| Wellbeing, m (SD) | 40.72 (8.85) | 39.62 (9.78) |
| School belongingness, m (SD) | 2.79 (0.67) | 2.86 (0.66) |
| Parenting: involvement, m (SD) | 29.58 (5.62) | 29.87 (6.35) |
| Parenting: positive parenting, m (SD) | 19.72 (3.63) | 19.67 (3.78) |
| Parenting: supervision, m (SD) | 17.35 (6.36) | 17.47 (6.42) |
| Parenting: inconsisent discipline, m (SD) | 15.52 (3.52) | 14.22 (3.68) |
| Parenting :punishment, m (SD) | 6.64 (2.52) | 6.59 (2.28) |
| Internalising caregiver report, m (SD) | 4.40 (2.19) | 4.52 (2.55) |
| Externalising caregiver report, m (SD) | 5.44 (2.72) | 4.93 (2.68) |
| Attentional problems caregiver report, m (SD) | 5.58 (2.26) | 5.53 (2.43) |
| Caregiver distress, m (SD) | 15.44 (5.36) | 14.47 (5.51) |

Table E. Summary statistics and results from mixed model analysis of primary and secondary outcomes for participants who

completed three-month assessment

|  | | Descriptive statistics | | Mixed model analysis | | |
| --- | --- | --- | --- | --- | --- | --- |
|  | Visit | Intervention | EUC | Difference in LS mean (95%CI) | P-value | Effect size^a^ |
|  |  | Estimated Mean (SE) | Estimated Mean (SE) |  |  |  |
| Primary Outcomes  Child Reported Outcomes | | | | | | |
| PSC Internalising | Baseline | 5.58 (.15) | 5.28 (.12) |  |  |  |
|  | 9-week | 2.96 (.14) | 3.34 (.12) | 0.69 (0.17, 1.20) | .01 | 0.37 |
|  | 3 months | 2.96 (.15) | 3.38 (.12) | 0.71 (1.19, 1.23) | .007 | 0.38 |
| PSC Externalising | Baseline | 7.26 (.20) | 7.38 (.16) |  |  |  |
|  | 9-week | 4.35 (.19) | 4.67 (.15) | 0.20 (-0.49, 0.89) | .57 | 0.08 |
|  | 3 months | 4.54 (.18) | 4.40 (.14) | -0.26 (-0.95, 0.44) | .46 | -0.10 |
| PSC Attention | Baseline | 5.58 (.15) | 5.56 (.12) |  |  |  |
|  | 9-week | 3.01 (.14) | 3.32 (.11) | 0.32 (-0.21, 0.85) | .24 | 0.16 |
|  | 3 months | 3.23 (.16) | 3.21(.13) | -0.01 (-0.55, 0.54) | .98 | -0.01 |
| PSC Total | Baseline | 32.40 (.67) | 33.08 (.55) |  |  |  |
|  | 9-week | 18.75 (.83) | 20.43 (.68) | 1.00 (-1.42, 3.43) | .42 | 0.11 |
|  | 3 months | 18.88 (.84) | 18.81 (.69) | -0.74 (-3.50, 2.02) | .60 | 0.12 |
| CRIES | Baseline | 24.47 (0.94) | 23.50 (0.76) |  |  |  |
|  | 9-week | 18.57 (0.64) | 18.05 (0.52) | 0.44 (-2.36, 3.25) | .76 | 0.04 |
|  | 3 months | 18.78 (0.72) | 18.90 (.58) | 1.08 (-1.91, 4.07) | .71 | 0.09 |
| PHQ-A | Baseline | 15.09 (.46) | 15.67 (.37) |  |  |  |
|  | 9-week | 12.92 (.36) | 12.27 (.29) | -1.23 (-2.72, 0.25) | .30 | 0.21 |
|  | 3 months | 12.40 (.35) | 12.34 (.28) | -0.65 (-2.19, 0.90) | .62 | -0.11 |
| ‏Functioning | Baseline | 16.88 (0.57) | 17.09 (0.46) |  |  |  |
|  | 9-week | 13.39 (0.50) | 17.34 (0.40) | 0.13 (-1.79, 2.04) | .89 | 0.02 |
|  | 3 months | 14.48 (0.352) | 14.37 (0.42) | -0.31 (-2.46, 1.83) | .77 | 0.04 |
| WEBWBS | Baseline | 40.81 (0.74) | 39.38 (0.60) |  |  |  |
|  | 9-week | 45.00 (0.56) | 44.89 (0.45) | 1.33 (-9.87, 3.64) | .26 | 0.14 |
|  | 3 months | 44.87 (0.60) | 45.11 (0.49) | 1.68 (-0.74, 4.10) | .17 | 0.18 |
| School | Baseline | 2.80 (0.05) | 2.84 (0.04) |  |  |  |
|  | 9-week | 3.11 (0.04) | 3.18 (0.04) | 0.02 (-0.14, 0.18) | .81 | 0.03 |
|  | 3 months | 2.98 (0.04) | 2.99 (0.03) | -0.03 (-0.20, 1.37) | .70 | 0.05 |
| Caregiver Reported Outcomes | | | | | | |
| Alabama Involvement | Baseline | 29.58 (.48) | 29.76 (.39) |  |  |  |
|  | 9-week | 30.47 (.43) | 30.62 (.36) | -0.02 (-1.58, 1.54) | .98 | 0.00 |
|  | 3 months | 30.69 (.43) | 30.49 (.35) | -0.37 (-1.94, 1.20) | .64 | 0.06 |
| Alabama Supervision | Baseline | 17.30 (.50) | 17.64 (.41) |  |  |  |
|  | 9-week | 17.60 (.48) | 16.47 (.39) | -1.46 (-3.12, 0.22) | .08 | 0.23 |
|  | 3 months | 16.82 (.43) | 15.78 (.35) | -1.38 (-3.15, 0.40) | .38 | 0.22 |
| Alabama Positive Parenting | Baseline | 19.75 (.29) | 19.57 (.24) |  |  |  |
|  | 9-week | 19.86 (.27) | 19.94 (.22) | 0.26 (-0.70, 1.22) | .59 | 0.07 |
|  | 3 months | 19.99 (.27) | 19.97 (.22) | 0.15 (-0.86, 1.17) | .77 | 0.04 |
| Alabama Discipline | Baseline | 15.48 (.29) | 15.36 (.23) |  |  |  |
|  | 9-week | 13.84 (.29) | 13.89 (.23) | 0.17 (-0.80, 1.14) | .73 | 0.05 |
|  | 3 months | 13.29 (.28) | 14.27 (.23) | 1.10 (0.10, 2.10) | .03 | 0.30 |
| Alabama Punishment | Baseline | 6.72 (.19) | 6.69 (.15) |  |  |  |
|  | 9-week | 6.19 (.16) | 5.72 (.13) | -0.45 (-1.04, 0.14) | .14 | 0.19 |
|  | 3 months | 5.74 (.15) | 5.76 (.19) | .05 (-0.55, 0.64) | .88 | 0.02 |
| PSC Internalising - Caregiver | Baseline | 4.41 (0.18) | 4.69 (0.15) |  |  |  |
|  | 9-week | 2.78 (0.18) | 3.08 (0.15) | 0.01 (-0.59, 0.61) | .98 | 0.00 |
|  | 3 months | 2.70 (0.18) | 2.67 (0.18) | -0.31 (-0.99, 0.36) | .36 | 0.13 |
| PSC Externalising - Caregiver | Baseline | 5.35 (0.17) | 5.06 (0.17) |  |  |  |
|  | 9-week | 3.92 (0.15) | 3.92 (0.15) | 0.70 (0.02, 1.39) | .04 | 0.26 |
|  | 3 months | 3.73 (0.16) | 3.73 (0.16) | 0.23 (-0.53, 0.99) | .56 | 0.08 |
| PSC Attention - Caregiver | Baseline | 5.54 (.18) | 5.65 (.18) |  |  |  |
|  | 9-week | 3.78 (.17) | 4.03 (.14) | 0.14 (-0.46, 0.74) | .64 | 0.06 |
|  | 3 months | 3.90 (.17) | 3.95 (.14) | -0.06 (-0.69, 0.57) | .85 | 0.03 |
| PSC Total - Caregiver | Baseline | 27.65 (.73) | 27.78 (.89) |  |  |  |
|  | 9-week | 21.18 (.73) | 19.91 (.87) | -1.39 (-4.15, 1.37) | .32 | -0.12 |
|  | 3 months | 19.08 (.71) | 19.31 (.86) | 0.11 (-3.27, 3.49) | .95 | 0.01 |
| K6 | Baseline | 15.34 (.40) | 14.30 (.33) |  |  |  |
|  | 9-week | 16.80 (.36) | 16.88 (.29) | 1.12 (-0.16, 2.40) | .09 | 0.21 |
|  | 3 months | 16.76 (.35) | 17.75 (.29) | 2.02 (0.74, 3.29) | .002 | 0.38 |

Abbreviations. EASE = Early Adolescent Skills for Emotions. EUC = Enhanced usual care. LS = Least Square. PSC = Paediatric Symptom

Checklist; CRIES + Children’s Revised Impact of Events Scale. WEBWBS = Warwick Edinburgh Mental Wellbeing Scale. PHQ-9A =

Patient Health Questionnaire Adolescent Version (total score range: 0-27; higher scores indicate more severe depression).  ^a^Effect size was

calculated by the difference in least square means between intervention and EUC from mixed model divided by the pooled standard

deviation at each visit.

Table F. Summary statistics and results from mixed model analysis of primary and secondary outcomes for participants

controlling for trauma exposure and postmigration stressors

|  | | | Descriptive statistics | | | Mixed model analysis | | |
| --- | --- | --- | --- | --- | --- | --- | --- | --- |
|  | Visit | Intervention | | EUC | Difference in LS mean (95%CI) | | P-value | Effect size^a^ |
|  |  | Estimated Mean (SE) | | Estimated Mean (SE) |  |  |  |  |
| Primary Outcomes  Child Reported Outcomes | | | | | | | | |
| PSC Internalising | Baseline | 5.58 (.14) | | 5.29 (.11) |  | |  |  |
|  | 9-week | 2.90 (.14) | | 3.33 (.11) | 0.69 (0.20, 1.18) | | .006 | 0.38 |
|  | 3 months | 2.96 (.15) | | 3.38 (.12) | 0.67 (0.17, 1.18) | | .008 | 0.37 |
| PSC Externalising | Baseline | 7.24 (.19) | | 7.34 (.15) |  | |  |  |
|  | 9-week | 4.36 (.18) | | 4.57 (.14) | -0.10 (-0.55, 0.75) | | .77 | 0.04 |
|  | 3 months | 4.54 (.18) | | 4.40 (.14) | 0.24 (-0.43, 0.91) | | .48 | 0.10 |
| PSC Attention | Baseline | 5.56 (.14) | | 5.54 (.12) |  | |  |  |
|  | 9-week | 2.97 (.14) | | 3.31 (.11) | 0.34 (-0.16, 0.84) | | .18 | 0.18 |
|  | 3 months | 3.23 (.16) | | 3.21 (.13) | -.03 (-0.55, 0.50) | | .93 | -0.02 |
| PSC Total | Baseline | 32.62 (.64) | | 32.47 (.52) |  | |  |  |
|  | 9-week | 18.44 (.80) | | 20.14 (.65) | 1.72 (-0.62, 4.07) | | .15 | 0.20 |
|  | 3 months | 18.93 (.84) | | 18.80 (.69) | -0.10 (-2.81, 2.60) | | .94 | -0.01 |
| CRIES | Baseline | 24.15 (0.87) | | 23.32 (0.70) |  | |  |  |
|  | 9-week | 18.38 (0.62) | | 18.24 (0.50) | 0.93 (-1.74, 3.59) | | .49 | 0.08 |
|  | 3 months | 18.78 (0.72) | | 18.89 (.58) | 1.18 (-1.69, 4.04) | | .42 | 0.15 |
| PHQ-A | Baseline | 15.07 (.43) | | 15.38 (.35) |  | |  |  |
|  | 9-week | 12.80 (.36) | | 12.37 (.29) | -0.76 (-2.22, 0.66) | | .29 | -0.13 |
|  | 3 months | 12.42 (.35) | | 12.36 (.28) | -0.38 (-1.87, 1.10) | | .61 | -0.06 |
| ‏Functioning | Baseline | 16.72 (0.54) | | 16.76 (0.43) |  | |  |  |
|  | 9-week | 13.28 (0.48) | | 13.68 (0.38) | 0.32 (-1.50, 2.13) | | .73 | 0.04 |
|  | 3 months | 14.53 (0.51) | | 14.44 (0.41) | -0.17 (-2.23, 1.88) | | .87 | -0.02 |
| WEBWBS | Baseline | 40.73 (0.69) | | 39.62 (0.56) |  | |  |  |
|  | 9-week | 45.08 (0.54) | | 44.96 (0.44) | 0.95 (-1.13, 3.14) | | .39 | 0.10 |
|  | 3 months | 44.87 (0.60) | | 45.11 (0.49) | 1.32 (-1.00, 3.64) | | .27 | 0.14 |
| School | Baseline | 2.79 (0.05) | | 2.86 (0.04) |  | |  |  |
|  | 9-week | 3.13 (0.04) | | 3.17 (0.03) | 0.04 (-0.12, 0.19) | | .66 | 0.05 |
|  | 3 months | 2.99 (0.04) | | 2.99 (0.03) | .07 (-0.10, 0.23) | | .42 | 0.12 |
| Caregiver Reported Outcomes | | | | | | | | |
| Alabama Involvement | Baseline | 29.58 (.45) | | 29.89 (.36) |  | |  |  |
|  | 9-week | 30.57 (.43) | | 30.71 (.35) | -0.13 (-1.61, 1.36) | | .87 | -0.02 |
|  | 3 months | 30.68 (.43) | | 30.50 (.35) | -0.44 (-1.95, 1.05) | | .56 | -0.07 |
| Alabama Supervision | Baseline | 17.35 (.47) | | 17.46 (.38) |  | |  |  |
|  | 9-week | 17.39 (.45) | | 16.46 (.37) | -1.07 (-2.63, 0.50) | | .18 | -0.17 |
|  | 3 months | 16.78 (.43) | | 15.74 (.35) | -1.19 (-2.88, 0.50) | | .17 | -0.33 |
| Alabama Positive Parenting | Baseline | 19.72 (.27) | | 19.67 (.22) |  | |  |  |
|  | 9-week | 19.81 (.27) | | 19.95 (.22) | 0.22 (-0.69, 1.13) | | .64 | 0.06 |
|  | 3 months | 19.97 (.27) | | 19.95 (.22) | 0.05 (-0.92, 1.02) | | .92 | 0.02 |
| Alabama Discipline | Baseline | 15.51 (.26) | | 15.23 (.21) |  | |  |  |
|  | 9-week | 13.69 (.28) | | 13.94 (.22) | 0.56 (-0.36, 1.49) | | .23 | 0.16 |
|  | 3 months | 13.29 (.28) | | 14.23 (.23) | 1.26 (0.30, 2.23) | | .01 | 0.35 |
| Alabama Punishment | Baseline | 6.64 (.18) | | 6.59 (.14) |  | |  |  |
|  | 9-week | 6.15 (.15) | | 5.69 (.13) | 0.41 (-0.15, 0.95) | | .15 | -0.17 |
|  | 3 months | 5.74 (.15) | | 5.75 (.12) | -0.05 (-0.63, 0.52) | | .85 | -0.02 |
| PSC Attention - Caregiver | Baseline | 5.58 (0.17) | | 5.54 (0.14) |  | |  |  |
|  | 9-week | 3.75 (0.16) | | 4.01 (0.13) | 0.34 (-0.24, 0.91) | | .25 | 0.15 |
|  | 3 months | 3.90 (0.16) | | 3.95 (0.13) | 0.12 (-0.50, 0.73) | | .71 | 0.05 |
| PSC Internalising - Caregiver | Baseline | 4.40 (0.18) | | 4.53 (0.14) |  | |  |  |
|  | 9-week | 2.79 (0.17) | | 3.02 (0.14) | 0.09 (-0.49, 0.66) | | .77 | 0.04 |
|  | 3 months | 2.69 (0.17) | | 2.68 (0.14) | -0.15 (-081, 0.51) | | .65 | -0.06 |
| PSC Externalising - Caregiver | Baseline | 5.44 (0.20) | | 4.93 (0.16) |  | |  |  |
|  | 9-week | 3.47 (0.18) | | 3.87 (0.14) | 0.91 (0.25, 1.56) | | .007 | 0.34 |
|  | 3 months | 3.78 (0.19) | | 3.73 (0.16) | 0.46 (-0.27, 1.19) | | .22 | 0.17 |
| PSC Total - Caregiver | Baseline | 26.98 (.70) | | 27.95 (.88) |  | |  |  |
|  | 9-week | 20.80 (.69) | | 19.60 (.84) | -2.26 (-4.95, 0.43) | | .10 | -0.19 |
|  | 3 months | 19.18 (.70) | | 19.33 (.86) | 0.90 (-4.23, 2.43) | | .59 | 0.07 |
| K6 | Baseline | 15.45 (.38) | | 14.46 (.31) |  | |  |  |
|  | 9-week | 16.90 (.35) | | 16.86 (.28) | 0.90 (-0.36, 2.15) | | .16 | 0.17 |
|  | 3 months | 16.75 (.35) | | 17.72 (.28) | 1.91 (0.66, 3.15) | | .003 | 0.37 |

Abbreviations. EASE = Early Adolescent Skills for Emotions. EUC = Enhanced usual care. LS = Least Square. PSC = Paediatric Symptom

Checklist; CRIES + Children’s Revised Impact of Events Scale. WEBWBS = Warwick Edinburgh Mental Wellbeing Scale. PHQ-9A

= Patient Health Questionnaire Adolescent Version (total score range: 0-27; higher scores indicate more severe depression).  ^a^Effect size was

calculated by the difference in least square means between intervention and EUC from mixed model divided by the pooled standard deviation

at each visit. All models controlled for trauma exposure and postmigration stressors.
