# Supplementary material for: Effectiveness of a brief group behavioural intervention on psychological distress in young adolescent Syrian refugees: A randomised controlled trial
Source: PLoS Med. 2022 Aug 12;19(8):e1004046. doi: 10.1371/journal.pmed.1004046 (PMC9374250; doi:10.1371/journal.pmed.1004046)
Supplement: S1 Text — (DOCX) [file pmed.1004046.s003.docx]

**RESEARCH PROTOCOL:**

**Improving Psychological Health in Adolescent Syrian Refugees in Jordan**

**(February, 2018)**

Prepared for University of New South Wales/ Al Basheer Hospital Ethics Committees

**PROTOCOL TITLE ‘Improving Psychological Health in Adolescent Syrian Refugees in Jordan’**

| **Short title** | **Improving Mental Health in Adolescent Syrian Refugees** |
| --- | --- |
| **Version** | **2** |
| **Date** | **February 10, 2018** |
| **Coordinating investigator/project leader** | ***Professor Richard Bryant*** |
| **Principal investigator(s)** | ***Professor Richard Bryant***  ***School of Psychology, UNSW*** |
| **Sponsor** | ***UNSW*** |
|  |  |
| **Subsidising party** | ***ELHRA*** |
|  |  |
| **Laboratory sites <*if applicable*>** | ***Not applicable*** |
|  |  |
| **Pharmacy <*if applicable*>** | ***Not applicable*** |
|  |  |

**SUMMARY**

**Rationale:** The current refugee crisis across the Middle East and Europe has large effects on individual refugees’ psychological wellbeing, as well as on the healthcare systems of countries housing refugees. The WHO have developed Early Adolescent Skills for Emotions (EASE), a brief (7-sessions), low-intensity psychological intervention, delivered by paraprofessionals, that addresses common mental disorders in people in communities affected by adversity.

**Objective**: The main objective is to evaluate feasibility, acceptability, and effectiveness of the culturally adapted EASE intervention for adolescent Syrian refugees in Jordan. The main hypothesis is that EASE will decrease psychological distress as compared to treatment as usual only. Psychological distress is operationalised as the subscale score on the Pediatric Symptom Checklist, including internalizing, externalzing, attentional, and total scors; this approach was adopted because EASE was developed specifically to mitigate internalising problems in young adolescents.

**Study population:** Young adolescent Syrian refugees (10-14 yrs) residing in Amman.

**Intervention (if applicable)**: Participants will be randomised to receive 7 sessions of EASE, an evidence-based psychological intervention aimed at reducing internalising problems in young adolescents, or treatment as usual (TAU). EASE is an evidence-based, low-intensity group intervention and will be delivered by trained providers in Jordan. The control group will receive TAU only.

**Main study parameters/endpoints:** The trial will be conducted with adolescents (aged 10 to 14 years) with high psychological distress and functional impairment will, after their caregiver and the adolescent has given consent and been enrolled in the study, be randomised to the treatment group and receive the EASE intervention or be allocated to the control group and receive enhanced treatment as usual. The primary measure for the participants is the Pediatric Symptom Checklist, which includes three subscales of internalising, externalising, and attentional problems, as well as a total score (Jellinek et al., 1999), and secondary measures include the Patient Health Questionnaire (PHQ-9) for adolescents (PHQ-A) (Johnson, 2002), post-traumatic stress symptoms (CRIES-13, Children and War Foundation, 2005), functioning (instrument to be developed), wellbeing (Short Warwick-Edinburgh Mental Wellbeing Scale, [SWEMWBS] Tennant et al., 2007), and sense of belonging and psychological engagement in school (Psychological Sense of School Membership [PSSM] scale). The caregivers of the young adolescent participants will also complete measures on psychological distress (Kessler 6, Kessler et al., 2002) and parenting (Alabama parenting questionnaire, (Shelton, 1996)). Exposure to traumatic events and parental involvement in the intervention will be assessed as potential moderators/mediators.

**Expected outcomes:** This project expects to provide evidence that EASE can reduce internalising problems in young adolescents when delivered by lay providers, as well as other psychological wellbeing benefits. **INTRODUCTION AND RATIONALE**

Recent crises in the Middle East, most notably in Syria, have resulted in an unprecedented increase in the number of refugees seeking asylum in neighbouring countries as well as in Europe. In 2015, over 1 million refugees have been registered entering Europe through the Mediterranean Sea (Refugees, 2015), and 4.8 million have fled to Syria’s neighbouring countries. Reports state that over 50% of Syrian refugees are children, in many cases unaccompanied by their family (UNHCR, 2016; UNICEF, 2016).

Refugees may have been exposed to multiple war stressors such including sexual violence and destruction of their homes and livelihoods, and they have often undertaken a risky and stressful flight leaving their homes for an unknown future. Studies show that refugees are at considerable risk to develop common mental disorders, including depression, anxiety, posttraumatic stress disorder (PTSD) and related somatic health symptoms (Steel et al., 2009).

Recent World Health Organization projections suggest that approximately 15-20% of Syrian refugees will develop some type of mental health issue (Hassan, 2015). Children refugees are especially at high risk for developing emotional problems, with a recent study in Syrian refugee children in Turkey reporting that nearly half of them show clinically significant levels of anxiety and withdrawal (Cartwright, 2015).

The refugee crisis imposes highly challenging demands on health systems in Europe and the Middle East. Given it adjacent position to Syria, Jordan hosts over 600,000 Syrian refugees. This has resulted in a sudden steep increase in numbers of individuals with mental health needs, and Jordan’s current health infrastructure is challenged to meet this need. In other countries in the Middle East and North Africa (MENA), such as Turkey and Lebanon, the mental health services required to meet the demands of millions of refugees in need are similarly inadequate and their health systems are overburdened to meet even basic survival needs and more chronic health problems (Gornall, 2015). The provision of services to address the psychological conditions of millions of refugees is conducted by non-government organisations (NGOs) and coordinated by international organizations, including the International Federation of Red Cross, United Nations High Commissioner for Refugees (UNHCR), International Medical Corps (IMC) and many others, including the Noor Al Hussein Foundation in Jordan.

The demand for scarce mental health services exceeds their availability in countries surrounding Syria, and there are also numerous barriers to the delivery and uptake of available services. Barriers to the delivery and uptake of mental health interventions for refugees include a lack of financial resources to pay for lengthy treatment programs, and limited capacity of mental health care specialists to deliver specialized services. In addition, the length and complexity of specialized treatments preclude simple access for refugees who are often unable to attend regular treatments over extended periods of time and to travel to attend sessions. Moreover, mental health programs usually focus on single psychiatric disorders (such as posttraumatic stress disorder; PTSD), whereas many refugees suffer multiple psychological problems that extend beyond single diagnostic boundaries (Thabet, Abed, & Vostanis, 2004; van Ommeren, Saxena, & Saraceno, 2005). Many refugees suffer from general psychological distress that does not need specialized mental health care interventions, but brief psychological interventions could be helpful to prevent more serious disorders. Finally, knowledge within refugee populations about mental health care supply is limited and stigma concerning mental health is prevalent in refugee populations and so many are reluctant to seek mental health care through formal services.

The World Health Organization has developed the low-intensity Problem Management Plus (PM+) programs, a new generation of shorter, less expensive and trans-diagnostic (i.e., not condition-specific) programs to reduce common mental health symptoms and improve psychosocial functioning. It is based on the WHO treatment guidelines for conditions related to stress (WHO, 2013). PM+ is a 5-sessions intervention that reduces symptoms of depression, anxiety, PTSD, and related conditions, is delivered by trained non-specialized workers or lay people, and is available in individual and group delivery formats (Dawson et al., 2015). It comprises evidence-based techniques: of (a) problem solving, (b) stress management, (c) behavioural activation, and (d) accessing social support. EASE has been successfully implemented in Kenya and Pakistan (Bryant et al., 2017; Rahman et al., 2016).

In a randomized controlled trial (RCT) in Kenya 410 women exposed to gender-based violence were randomized to PM+ or enhanced treatment as usual (TAU). Local volunteer health workers with no mental health experience were trained in EASE , and delivered 5 sessions of PM+ to each participant. Relative to TAU at a three-month follow-up assessment, PM+ resulted in greater reductions in anxiety and depression, posttraumatic stress, and functional disability (Bryant et al., 2017). Similarly, a controlled trial of 344 people in Pakistan affected by terrorism and war found that those who received five sessions of PM+ had greater reductions in anxiety, depression, functional disability, and posttraumatic stress than those who received an enhanced treatment as usual (Rahman et al., 2016). As a result, the WHO has adopted PM+ as the key low-intensity mental health intervention approach to be globally implemented within primary and community health care to populations affected by adversity.

In the wake of the success of this program, UNSW in collaboration has developed the EASE program, which is a variant of PM+ developed for young adolescents. This project aims to conduct the initial trial of this protocol by testing it with young adolescent Syrian refugees in Jordan. The principal investigator of this study, Professor Richard Bryant, led the study in Kenya and was a co-investigator in the Pakistan trial.

**Development of a new WHO intervention for young adolescents**

The intervention called Early Adolescent Skills for Emotions (EASE) has been devised to reduce internalising problems in young adolescents, and can be delivered by trained non-specialized providers or lay people, and is delivered using a group format (see text box below). EASE is comprised of 7 group sessions for the young adolescents that use evidence-based techniques including: psychoeducation, stress management (slow breathing), behavioural activation, problem solving and relapse prevention. There are also three group sessions for the caregivers and the following discrete strategies include: psychoeducation, active listening, stress management (slow breathing), quality time, praise and showing interest in their child, caregiver self-care and relapse prevention for their child. The involvement of parents/caregivers is supported by the recently revised WHO mental health Gap Action Programme (mhGAP) intervention guidelines, which recommend caregiver skills training for emotional and behavioural disorders in children (WHO, 2016).

The process of development of EASE is provided in Text Box 1 below. The intervention consists largely of cognitive and behavioural strategies, which have generally shown effectiveness in treating anxiety (James, James, Cowdrey, Soler, & Choke, 2015), post-traumatic stress (Gillies, Taylor, Gray, O'Brien, & D'Abrew, 2012) and depression (Haby, Tonge, Littlefield, Carter, & Vos, 2004). The intervention development involved several steps of detailed peer-review from experts in child psychotherapy.

EASE has four core features, that are unlike any other programmes. It is:

- Brief (7 group sessions for the young adolescents and 3 group sessions for their caregivers),
- Delivered by non-specialised providers,
- Transdiagnostic, addressing depression, anxiety, distress and problems as defined by the young people themselves, and
- Designed for young people and their caregivers in low and middle-income countries living in communities affected by adversity (e.g. armed conflict)

| Development of EASE (by WHO) *Background*  Adversity is a risk factor for the development of anxiety and depression. WHO recommends the use of evidence based psychological interventions for the treatment of emotional conditions in children and adolescents. However access to such treatments in low- and middle-income countries (LMICs) is limited. WHO’s programme for the development of scalable psychological interventions aims to publish a range of scalable psychological intervention manuals for populations across the lifespan within communities affected by adversity.   - “Communities affected by adversity” refers to communities where many people have experienced extreme stressors through single events such as a natural disaster or an act of terrorism, or through more sustained adverse conditions, such as chronic poverty, endemic community and interpersonal violence or long-term civil conflict or displacement. - “Scalable” intervention means a *low resource intensity* intervention. Characteristics that make an intervention low resource intensive include; (1) being a brief or modified version of an evidence-based therapy; (2) possibly being transdiagnostic; (3) having a primary focus on teaching skills to an individual or caregiver for the management of common mental health problems, (4) adaptability to different modalities such as guided self-help or e-mental health, and (5) is deliverable by para-professionals, requiring limited input from specialists.   *Process of development*   1. A scoping review was conducted which reflected findings from systematic reviews reporting low to moderate effect sizes for psychological interventions with children and adolescents affected by adversity in LMICs. 2. Evidence to inform development of the intervention was identified from a component analysis of published effective psychological interventions from mainly high income countries for two age ranges, 10-14 and 9-15 years. The following seven intervention components were in the top 10 of both age-range searches: cognitive, problem solving, relaxation, exposure, psychoeducation for children, modelling, and maintenance/relapse prevention.   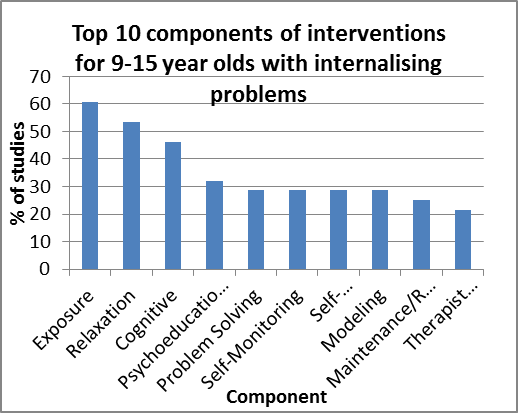   1. Consultations with 13 experts provided further guidance for the intervention components with problem solving, skills to promote relaxation and emotion regulation, activities to enhance social interactions, cognitive approaches and psychoeducation being commonly recommended components. Advice was also provided for factors related to intervention delivery (e.g. age range, parental involvement, flexibility of intervention) and therapeutic techniques (e.g. use of storytelling, group activities, skill rehearsal). |
| --- |

# Current Project Objectives

This project is seeking to address the high burden of disease associated with internalising, externalising, and attentional symptoms through a potentially cost-effective and scalable evidence-based intervention for young adolescents. We seek to conduct a pilot evaluation and definitive RCT of a new brief group psychological intervention for young adolescents in Lebanon. There will be a focus throughout on determining whether the intervention is suitable to address the mental health needs of Syrian adolescent refugees

# Methodology

### Participants and procedure

**Inclusion and exclusion criteria**

### *Inclusion Criteria:*

- Aged between 10 and 14 years
- Syrian adolescent refugees
- Able to follow and understand instructions
- Has a caregiver that has provided consent for participation (NOTE: there is no inclusion/exclusion for the caregiver, a caregiver is any willing adult that is defined as the primary caregiver by the young adolescent. This may be their parent or other family member (e.g. grandparent/aunty).
- Maximum one child per family will be included. In situations where more than one eligible child is identified within the family (e.g. two siblings, both screened positive), the eldest child will be offered the opportunity to participate. In situations where the caregiver and or the child remain concerned about their psychological distress and functioning, they will be encouraged to seek psychosocial support through locally accessed community support, via Noor Al Hussein programs.

*Exclusion criteria:*

- Unaccompanied minors
- Children or caregivers that are at risk for harming self and/or others
- Children or caregivers that unable to understand explanations instructions
- Imminent risk of child abuse and requires urgent child protection
- Imminent suicidal risk
- Severe mental disorders

### Statistical Power and Sample Size

The required sample size was calculated on the basis of the estimated sample size required in the control arm, considering the number of EASE groups, the number of participants in each group with data at the 3-month follow-up time point, the effect size, the ratio of variance in the EASE arm versus the control arm, and the intracluster correlation coefficient. We conservativly estimated a theta of 1.1 with an intraclass correlation of 0.13. We estimated that with 20 EASE groups (at least 6 people per group) each at the 3-month primary outcome timepoint (and assuming a 5% two-tailed significance test and 80% power), data from 191 participants in the control arm would be needed at the 3-month follow-up for a moderate effect size (0.4). These calulations correspond to an sample size of 311 at 3-months, and an allocation ratio of EASE to EUC arms of 1:1.6. We projected attrition 33% loss to follow-up, we estimated the sample size required at enrolment would be approximately 470.

**Recruitment Process**

Potentially eligible adolescents will be recruited by household screening in Amman (an area that is heavily populated by Syrian refugees). To obtain the required sample size, assessors will approach consecutive households within the designated area. Assessors will seek to screen one child per household.

### Screening and consent procedure

See Figure 1 for a study flowchart. Caregivers of all children and adolescents (aged 10 to 14 years) reached through above-mentioned outreach activities will initially be asked for consent for their child to be invited to undertake a screening procedure using the Paediatric Symptom Scale (PSC-17 (Jellinek et al., 1999) upon which a score of scored ≥15 will be required to indicate distress. The child will also then be invited to give their assent in completing the screening measure. The PSC is a 17-item screening measure of generic child psychological distress. Higher total scores indicate higher levels of psychosocial distress. Ineligible children (and their caregivers) will then be advised of the outcome of the screening measure and of their inability to participate in the study. The children and their caregivers will receive education materials on how to cope with difficult situations. In situations where the caregiver and or the child remain concerned about their psychological distress and functioning, they will be encouraged to seek psychosocial support through locally accessed community support, which will be facilitated via the Institute of Family Health programs. Eligible children based on the results from the PSC, and their caregivers, will then be invited to participate in the research study. At this point, detailed information about the study will be provided and applicants invited to take part, with caregivers required to provide consent first followed by their child’s assent. Once parental consent and child assent are obtained, the child will be enrolled in the study and baseline measurements will be conducted.

Given potential illiteracy and varying developmental stages of young adolescents and their caregivers, the consent form will be read in local, lay language to all caregivers and participants. After providing verbal consent, literate participants and caregivers will be asked to acknowledge the process with a signature. For participants and their caregivers with literacy difficulties, a witnessed thumb print in lieu of a signature will be sufficient. It will be ensured that potential participants and their caregivers fully understand what it means to participate and that they can withdraw their consent at any time without having to give an explanation. It will also be made clear that refusal to participate will not have an impact on any type of support they receive.

Figure 1: Study Flowchart
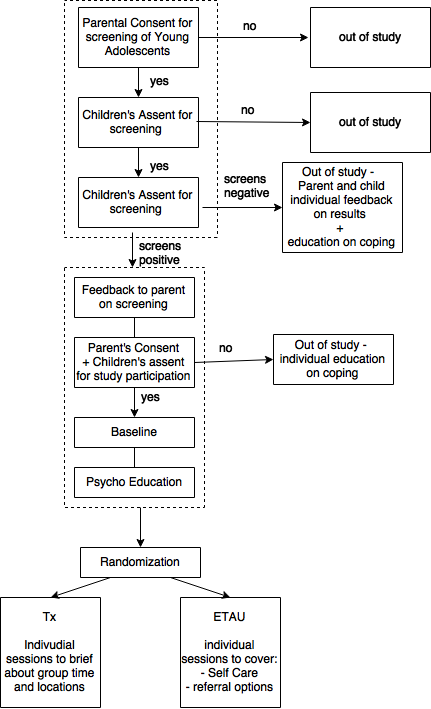


**Instruments**

Primary and secondary outcome measures will be completed with the participants and their caregivers at three time points: pre (upon enrolment to the study/ week 1), post (at completion of the treatment/ week 8), 3-month follow-up (primary timepoint), and 12 month follow-up after the completion of the treatment/ week 52 [secondary timepoint]). The primary outcome point is the 3-month assessment. See Table 1 below for details of the tools to be used – with relevant psychometric properties included. All tools are currently available in Arabic, and where locally appropriate language versions are not available, measurement translation will occur alongside intervention translation. The primary outcome measure will be the PSC-35, including the 3 subscales of Internalising, Externalising, and Attentional, as well as the total of the PSC. This approach is indicated because the EASE program is intended to reduce internalising problems, however the project also measures the extent to which externalising, attentional, and overall psychological problems are impacted (as measured by the PSC).

In addition to the selected outcome measure potential moderators will be assessed, including socio-demographic variables, exposure to traumatic events and level of parental involvement in the intervention.

Table 1**:** Measures to be used in the young adolescent intervention

|  | **Domain** | **Tool** | | **Number of items** | **Psychometric properties** | **Timing** |
| --- | --- | --- | --- | --- | --- | --- |
|  |  | |  |  |  |  |
|  | - General child psychological distress | Pediatric Symptom Checklist-17 | | 17 | AUC = .95 (Good cross cultural construct validity) | Screening; |
|  |  | |  |  |  |  |
|  | Primary |  | |  |  |  |
|  | - Internalising, externalising, attentional, general distress | Pediatric Symptom Checklist – youth version (Jellinek et al., 1999) | | 35 | AUC = .92 (lower income sample) | Pre- intervention  1-week Post  3- month Post  12-month Post |
|  | Secondary |  | |  |  |  |
|  | - Depression | Patient Health Questionnaire 9 for adolescents (PHQ-A; (Johnson, Harris, Spitzer, & Williams, 2002) | | 9 | AUC = .88 (for adolescents) | Pre- intervention  1-week Post  3- month Post  12-month Post |
|  | - Posttraumatic stress | The Children’s Impact of Events Scale (CRIES-8; (Perrin, Meiser-Stedman, & Smith, 2005) | | 8 | AUC = .80 | Pre- intervention  1-week Post  3- month Post  12-month Post |
|  | - Involvment in school | Psychological Sense of School Membership Scale (Goodenow, 1993) | | 18 | Chronach’s alph = 0.87 | Pre- intervention  1-week Post  3- month Post  12-month Post |
|  | - General functioning | Impairment in daily functioning | | 10 | -- | Pre- intervention  1-week Post  3- month Post  12-month Post |
|  | - Well-being | Short Warwick-Edinburgh Mental Wellbeing Scale (SWEMWBS; (Tennant et al., 2007) | | 14 | Cronbach's alpha = .89; TRT ICC = .83 (among teenagers) | Pre- intervention  1-week Post  3- month Post  12-month Post |
|  | **Caregiver** | |  |  |  |  |
|  | - General psychological distress | Kessler 6 (K6; (Kessler et al., 2002) | | 6 | Cronbach's alpha = .89 - .92;  AUC = .88 | Pre- intervention  1-week Post  3- month Post  12-month Post |
|  | - Parenting | - Alabama Parenting Scale (Shelton, 1996) | | 42 | Cronbach's alpha > .70; TRT *r* = .84 - .90 | Pre- intervention  1-week Post  3- month Post  12-month Post |
|  | - Exposure to traumatic events | Harvard Trauma Questionnaire (HTQ) (Mollica, Caspi Yavin, Bollini, Truong, & et al., 1992) | | 17 | Cronbach's alpha = .92; TRT Cohen's kappa *k* = .64 (for adolescents) | Pre-intervention |

Note: AUC – Area Under the Curve; TRT = Test Retest Reliability; ICC = Intra Class Coefficient.

**Interventions**

**Enhanced Treatment as Usual (ETAU)**

The control group will receive enhanced treatment as usual (ETAU). This will involve a single psychoeducation session, jointly for eligible children and their caretakers, that includes information (i) about the results of the screening, (ii) self-care strategies and (iii) seeking services from local health or community services offering psychosocial/ mental health care support. In situations where the caregiver and or the child remain concerned about their psychological distress and functioning, they will be encouraged to seek psychosocial support through locally accessed community support. Any referrals will be conducted in line with the national standards and procedures for individual referrals in Jordan.

### EASE Intervention

The Early Adolescent Skills for Emotions (EASE) intervention program is a new, brief, group psychological intervention program based on cognitive behavioural therapy (CBT) techniques that are empirically supported and formally recommended by the WHO (WHO, 2016). The full protocol was developed by WHO and the University of New South Wales, Australia. The intervention is comprised of 7 group session lasting 90 minutes for the young adolescents and is accompanied by 3 group sessions for their caregivers, each lasting 120 minutes. The young adolescent sessions involve the following empirically supported components: psychoeducation, problem solving, stress management (slow breathing), behavioural activation, and relapse prevention. The caregiver sessions involve psychoeducation, active listening, quality time, praise, caregiver self-care and relapse prevention. Figure 2 shows a brief outline of the timeline for the seven sessions for the young adolescents and the three sessions for their caregivers.

Figure 2. Outline of the Timeline for the EASE Sessions

| **Time line** | **Session** |  |
| --- | --- | --- |
| Week 1 | Caregiver Assessment Session Youth Assessment Session |  |
| Week 2 | Caregiver Session1 | Understanding my feelings |
|  | Youth Session 1 | Understanding Sadness, Worry and Stress |
| Week 3 | Caregiver Session 2 | Calming my body |
|  | Youth Session 2 | Boosting Confidence |
| Week 4 | Caregiver Session 3 | Getting active |
|  | Youth Session 3 | Caregiver Self-care & Brighter Futures |
| Week 5 | Youth Session 4 | Getting active |
| Week 6 | Youth Session 5 | Stop, Think, Go |
| Week 7 | Youth Session 6 | Stop, Think, Go |
| Week 8 | Youth Session 7 | Brighter futures |
| Week 9 | Caregiver Post Assessment Youth Post Assessment |  |

**Study Procedures and Randomization**

**Randomization**

After participants complete baseline assessments, they will then be randomised into the treatment group or enhanced treatment as usual (ETAU; control). The randomisation sequence will be computer generated with a 1:1.6 allocation using random block sizes of two and four. Treatment allocations will be recorded on pieces of paper, which will be folded and placed inside sealed, numbered, opaque envelopes by a staff member not involved in the study. Allocation to groups will be conducted after baseline assessments have been conducted. Envelopes will be opened by the implementation team, as each participant completes baseline. Given the nature of the study, participants and facilitators will not be blinded to group assignment. Assessors completing the baseline and follow-up assessments will be blind to group assignment and therefore will not be involved in the randomization process. After randomization, individual feedback will be provided to the participants and their caregivers as to their group allocation. For those randomised into the treatment group, details about when and where the intervention sessions will commence will be provided. For those randomised to the control group, the ETAU session will be scheduled.

Compensation to participate

Gift reimbursement for transportation of caregivers and children will be given gifts for assessment based on the rate of $JD3 per assessment. Refreshments will also be provided during group sessions. Reimbursement is not provided for attendance at intervention sessions.
Ensuring blinding

Blinding of assessors will be conducted by ensuring that assessors and facilitators work in separate offices and are not in contact. Assessors will prompt participants to not share any information on the type of treatment that they receive and explain that they are not supposed to know. Before assessment assessors will be asked to indicate if they think participants are allocated to the control or intervention condition. This will provide some data of the amount of unblinding that might occur in the RCT.

### Participants who discontinue the intervention

Telephone follow-up will be attempted (up to three times) if a participant and or/ their caregiver does not attend their group session. Participants will be invited to discuss why, and reminded that this is a voluntary programme. If participants indicate that they wish to discontinue the intervention they will be invited to provide the non- specialised provider with a reason for discontinuation, and will be reminded that this is not required. The purpose of asking these questions is to maximize retention and to learn about barriers for participation. If participants express that they discontinued the intervention due to heightened distress, appropriate alternative referrals will be discussed with the participant and initiated when necessary.

### Recruitment, Training and Supervision of Facilitators

### Recruitment

EASE providers will be male and female non-specialised providers with a qualification in health service or related discipline. They will be recruited though an open vacancy process, and go through a process of interviews to select the group who will be invited to receive training. After termination of the training, all participants will do an assessment of competencies through a standardized role-play in order to select the group of facilitators who will be invited to implement the trial.

**Training**

EASE facilitators will receive two weeks (60 hours) of training. Intervention training includes education on adversity and its impact upon mental health, basic counselling skills, delivering EASE, skills in group facilitation, and facilitator self-care. Training of providers will be conducted by master trainers who have been involved in the development of the EASE protocol. In addition, all intervention providers will receive a 1 day training in psychological first aid especially to deal with crisis situations (WHO, War Trauma Foundation and World Vision International, 2011).

**Supervision**

EASE trained psychologists will be responsible for supervising the EASE providers. The team of supervisors will consist of May Aoun (Lebanon, War Child), Katie Dawson (Australia, UNSW), and Aiysha Malik (Switzerland, WHO). Weekly supervision will be provided to EASE providers by an appropriately qualified, clinical supervisor with a good understanding of the young adolescent project to ensure fidelity of guidance provided, and to support helpers; she will be supported by clinical supervisors who have been involved in the development of EASE (Katie Dawson and Aiysha Malik). Supervision will involve discussion of difficulties encountered in supporting the users of the intervention, as well as self-care for the staff. This also forms an integral part of continued learning (e.g. through role-plays and associated teaching methods).

### Treatment protocol adherence and quality control

After training is conducted with potential EASE providers a competency test will be conducted to assess the knowledge of training attendees. The competency test will make use of the Enhancing Assessment of Common Therapeutic factors (ENACT) rating scale for training and supervision. The ENACT scale is an 18-item assessment for common factors in psychological treatments, including task-sharing initiatives with non-specialists across cultural settings. Trainees that pass the ENACT scale will become EASE providers in the pilot. Protocol adherence will be ensured by the supervisors and weekly supervisions of the EASE non-specialised providers (Murray et al., 2011). A checklist will be developed to assess a random sample of audio-taped sessions of 10-15% of sessions for each facilitator for treatment fidelity. In case the use of audio devices is not accepted by many participants of the exploratory trial, we will change this part of the protocol; instead, research assistants (other than those that are included in the outcome measurements, to ensure keeping research assistants blinded to the treatment arms) will be present during the EASE sessions to code treatment fidelity on a checklist. Process indicators will be collected through monitoring and the supervision structure. Random fidelity and competence checks will be conducted by supervisors as part of their supervision to verify the delivery of EASE to participants. ETAU adherence will be measured through assessments of attendance at mental health services.

### The Jordan setting

Jordan was selected as the setting for adaptation and feasibility testing for various reasons. In Jordan, Noor Al Hussein and UNSW are well placed in being the main project partners. This collaboration of public health, research and field-based health care provision organisations will build on existing strong partnerships and provide the necessary knowledge and skills for intervention adaptation and piloting the delivery model.

### Delivery of the intervention in Jordan

The delivery model has been developed to address problems with Jordan’s limited mental health workforce and its low coverage of specialist services. The intervention has been designed to reduce the amount of time health care workers spend with clients each week and reduce the number of referrals to specialists for psychological interventions. If the intervention does not lead to resolved symptoms, users can proceed to the next step of care (e.g. an individual intervention). For the EASE intervention pilot, there are a number of specifications that participating facilities must meet to ensure user safety and the research integrity of the pilot:

- The community facilities must be able to organise care for young adolescents identified as being in need of further mental health support during the pilot (e.g. availability of specialists or staff trained in mhGAP, or a functioning referral network to other services);
- Availability of staff who provide weekly guidance to users throughout the intervention (e.g. case managers, family doctors, social workers, nurses or community care providers);
- Facilitator staff who deliver the intervention must be available to receive supervision in delivery of the intervention.

### Risk mitigation phase

Since the young adolescent intervention is non-pharmacological and there is a broad evidence base for its safe use, it is unlikely that adverse effects due to the intervention will occur in the pilot study. The face to face contact with the trained facilitator will ensure psychological distress is monitored at each week. There will be an independent safety monitoring board to consider adverse reactions. We plan to closely monitor the safety of the intervention in this controlled pilot, prior to testing the intervention in an RCT

### Participants and procedure

### Data Management and Statistical Analysis

A password protected tablet will be used to collect data by independent assessors. Kobo Toolbox software will be used for the Data Collection. The data on the tablet will be synchronized and uploaded first on the Kobo Toolbox Server. The server is an Microsoft Azure server, which is based in West-Europe (cloud-based). From Jordan the Research Coordinator will have access to a limited part (only accessible by primary, co-investigators) in order to store the datasets before data transformation and analysis will take place. In Sydney (UNSW), the datasets will be uploaded subsequently on another part of the Azure server. All study laptops are encrypted and password protected. UNSW Data Manager will control the access to this office. The data is accessible on this server during the life of the particular trajectory and will remain accessible in an archive until 7 years after completion of the study.

**Quantitative data collection**

Quantitative data will be coded and the identifying key (a list connecting names to numbers) will be kept in a separate, secure locked location in the coordinating researcher’s office (Mr Aemal Akhtar). The data will be entered into a data-analytic computer program (e.g., SPSS), without the identifying key. Data will only be available to the members of the project group. No attributable data will be used in publications.

**Data Analysis Plan**

The data will be analyzed on an intention-to-treat and completers analysis. Quantitative characteristics are reported using means and standard deviations (or medians and interquartile ranges) and categorical characteristics using percentages. We report summaries for the baseline characteristics of each trial arm. Primary analyses focus primarily on intention-to-treat. We will use linear mixed models to study the effects of the treatment arms. Fixed (intervention, time of assessment) effects and their interactions will be included, which provides an index of the relative effects of the treatments; and time of assessment including baseline, posttreatment, and 3-month follow-up. Primary analyses will focus on the PSC subscales and total score because EASE was designed to target internalising problems. The secondary outcomes (PHQ total scores, PHQ-A, CRIES-13, WEMWBS, PSSM, K6, APQ, PSC caregiver report) will be analysed with the same analytic approach. We will also conduct sensitivity analyses with participants who complete the 3-month follow-up. To determine the efficacy of the intervention on participants with more severe psychological disorders, we will also conduct secondary analyses focusing on adolescents who scored ≥5 on the PSC internalising subscale. Exposure to traumatic events and parental involvement in the intervention will also be assessed as potential covariates/moderators of outcomes. Secondary analyses will also be conducted at the secondary time point of 12 months follow-up. Findings will be reported according to the CONSORT guidelines for randomized controlled trials. Data will be stored for a period of at least 7 years. Results of the data will be published regardless of being negative or positive results and submitted to peer- reviewed scientific journals.

# Project Management

# Safety Considerations

Participants in both arms will have access to trained health staff in the districts. When necessary they will be referred to a specialist for further assessment for any identified child protection services or management of severe psychiatric problems. All referrals will be conducted in line with the national minimum standards and procedures that are consistent with child protection services in the Institute of Family Health.

# Adverse Events Reporting Mechanism

We will monitor the occurrence of specific serious adverse events using the an Adverse Events Reporting Procedure. Death, suicide attempt, emergency psychiatric hospitalization, side-effects of impairing function, domestic violence, sexual abuse, fainting during data collection, violence to researcher and others– will be monitored. All adverse events will be reported to a data safety monitoring board (DSMB). This will occur within 24 hours. If necessary, appropriate action in respect of ongoing trial conduct will be taken (such as referral to specialized care or installing extra assessment points for monitoring participants). We consider an event an adverse event if it is an undesirable experience occurring to a subject during the study, whether or not considered related to the research procedure. Although it is unlikely that Adverse Events would occur given the nature of the intervention, all Adverse Events will be reported to the WHO Ethical Review Committee, and the local ethical committee in Jordan.

With specific regard to indications that any child is experiencing physical or sexual abuse during the course of the study, the child safety and reporting mechanisms of the Institute of Family Health will be implemented to ensure that all reporting and child-protection procedures are appropriately followed. Depending on the event, follow up may require additional tests or medical procedures as indicated, and/or referral to a general physician or a medical specialist. All adverse events will be followed until specialist care (including referrals, additional tests or medical procedures) is in place for the client, or until a stable situation has been reached.

On the informed consent form, participant information is included to inform participants that research staff, or another clinician other than their facilitator are available to them if they are upset by this study.

Research assistants completing the assessments and facilitators completing the intervention, will be rigorously trained in sensitive interviewing techniques, responding to distress, and protocols to follow in the unlikely event of significantly increased distress. Before being deployed all staff will be made aware about the potential stressful nature of the job and will receive information on self-care strategies. During the project, the research assistants and facilitators will also get support and supervision to handle possible difficulties they might experience.

# Ethics

Ethical considerations include the following

- This study includes adults and children younger than 18 years of age (minors are involved). Previous studies using similar methodologies (e.g. PM+ in Pakistan and Kenya, Group PM+ in Pakistan and Nepal, SH+ in Uganda) have been fully approved by international and local review committees. This application follows the ethics submission to WHO’s Ethical Review Committee made by War Child Holland regarding a parallel trial of EASE being conducted with young adolescents, including Syrian refugees, in Lebanon. Ethical approval to conduct the study is being gained from the University of New South Wales (Sydney) and the Ethics Committee of Al Basheer Hospital (Amman). The informed consent procedure used in this study is described in this protocol and follows recommendations by WHO’s Ethical Review Committee (<http://www.who.int/rpc/research_ethics/Process_seeking_IF_printing.pdf>).
- This procedure includes: (1) oral and written information to consider participation; (2) a variant for illiterate participants, who may give consent through both a signature of a literate witness (not a member of the research team) and a thumb print. Full information on the study will be provided in local, lay language before obtaining consent (written or oral as described above) from each participant. To participants who are illiterate the information will be read out in the presence of an independent witness not affiliated to the study. It will be ensured that potential participants fully understand what it means to participate and that they can withdraw their consent at any time without having to give an explanation. It will also be made clear that refusal to participate will not have an impact on any type of support they receive.
- EASE is a new intervention and an exploratory RCT is necessary to establish safety and acceptability (Craig et al., 2008). Participants in the control condition will not receive EASE, instead will receive a 1-session psychoeducation intervention, with the opportunity to be referred to more specialized care in case of detected need. Given that currently most young adolescents in Jordan will not have access to mental health services offering the possibility for referral is considered enhanced treatment as usual
- We seek to ensure that involvement in the intervention and study does not stigmatize participants in any way, through the following mechanisms: (a) it is a community-based intervention, delivered in the community rather than at a facility; (b) it is trans-diagnostic, so does not target a specific type of disorder (the intervention is specific to the broad category of common mental disorders); (c) the aim of EASE is to be non-pathologizing, it does not involve identifying diagnostic categories and the manual includes no medical terminology; and (d) stigmatizing and medicalising words associated with mental disorders are not used in the intervention.
  The research team has an extensive background in appropriate adaptation of instruments, consent forms, recruitment material, and other study related resources to minimize stigma and optimize mental health benefit to Lebanese participants.
- The research team is considerate of the fact that the study will be conducted with vulnerable young adolescents that are exposed to adversities and experiencing psychological distress. However, the risks for participants in EASE (i.e. young adolescents impaired by distress) are considered minimal. EASE is expected to have a therapeutic benefit and reduce symptoms of distress and common mental disorders, improve quality of life, and improve well-being. The training and EASE manual contains guidelines on how to deal with adverse events or possible persistent or worsening symptoms that may occur. The implementation packages also include training on supervision, and the fidelity of how EASE is used will be ensured by doing competency assessments. Facilities are in place to monitor this and appropriate actions will be taken based on an established crisis assessment protocol (for instance, referral to more intensive psychological treatment and monitoring of participant by research staff).
- It is essential to conduct this research in a vulnerable population and with this age group, because rigorous evidence needs to be collected on the effectiveness of this intervention especially developed for this vulnerable population. Additionally, it will provide valuable information about how to adapt the intervention and other important considerations for scale up.
- Participants will receive an equivalent of $JD3 per assessment. Assessments will take a maximum of one hour and thirty minutes and participants will be informed of this time frame as part of the consent process. Participants will not be compensated monetarily for the time they spend on the sessions. During the EASE trial, snacks and drinks will be offered to the participants at every session. Travel costs to assessments and sessions will also be compensated for. Actual cost basis is not currently feasible because of the unavailability of local transport receipts, but a fixed amount for compensation will be calculated based on the area that they come from.

# Quality assurance

Participants in this study may directly benefit from their participation in the intervention, which is based on techniques that are empirically supported. Their participation will furthermore inform local adaptation of such materials, and thus improve knowledge about delivering mental health interventions in the study area.

# Expected Outcomes of the Study

It is expected that the current study will increase knowledge about the feasibility, acceptably, effectiveness and potential for wider scale-up of EASE with adults exposed to adversities in low resourced settings. It has the potential to provide valuable information for context-specific interventions (like EASE) to be further adapted and rolled out in other settings. By testing EASE in a variety of real-life contexts, information will be collected on the requirements that should be in place to successfully implement an intervention like EASE.

Insight into these minimal requirements can be used to further scale up task-shifting interventions in humanitarian settings to address the mental health and psychosocial needs of affected populations. WHO will have enough evidence to release the EASE manual after two RCTs showing positive results. In this way it can potentially help to reduce mental suffering and improve the well-being and functioning of young adolescents affected by armed conflicts and disasters. Adequate ability to function can be essential for survival and is essential for socio-economic functioning of survivors and their communities and therefore this intervention can have a significant impact on many lives.

# Dissemination of Results and Publication Policy

The results of this project will be published in English in peer-reviewed journals (we will aim to publish in open access journals). It will be disseminated in Arabic and English to key stakeholders (like heads of relevant services, participants in community engagement meeting) by reports and presentations. District, provincial and national government and health authorities will be made familiar with the results of the project and a dissemination workshop for local health workers and policy makers will be organized.

# References

Bryant, R. A., Schafer, A., Dawson, K. S., Anjuri, D., Mulili, C., Ndogoni, L., . . . van Ommeren, M. (2017). Effectiveness of a brief behavioural intervention on psychological distress among women with a history of gender-based violence in urban Kenya: A randomised clinical trial. *PLoS Med, 14*(8), e1002371. doi:10.1371/journal.pmed.1002371

Cartwright, K., El-Khani, A., Subryan, A., & Calam, R. . Global Mental Health 2015; 2. (2015). Establishing the feasibility of assessing the mental health of children displaced by the Syrian conflict. *2*.

Craig, P., Dieppe, P., Macintyre, S., Michie, S., Nazareth, I., Petticrew, M., & Medical Research Council, G. (2008). Developing and evaluating complex interventions: the new Medical Research Council guidance. *BMJ, 337*, a1655. doi:10.1136/bmj.a1655

Dawson, K. S., Bryant, R. A., Harper, M., Kuowei Tay, A., Rahman, A., Schafer, A., & van Ommeren, M. (2015). Problem Management Plus (PM+): a WHO transdiagnostic psychological intervention for common mental health problems. *World Psychiatry, 14*(3), 354-357. doi:10.1002/wps.20255

Gillies, D., Taylor, F., Gray, C., O'Brien, L., & D'Abrew, N. (2012). Psychological therapies for the treatment of post-traumatic stress disorder in children and adolescents. *Cochrane Database Syst Rev, 12*, CD006726. doi:10.1002/14651858.CD006726.pub2

Haby, M. M., Tonge, B., Littlefield, L., Carter, R., & Vos, T. (2004). Cost-effectiveness of cognitive behavioural therapy and selective serotonin reuptake inhibitors for major depression in children and adolescents. *Aust N Z J Psychiatry, 38*(8), 579-591. doi:10.1080/j.1440-1614.2004.01421.x

Hassan, L. J., Mekki-Berrada, A., Quosh, C., el Chammay, R., Deville-Stoetzel, J.B., Youssef, A., Jefee-Bahloul, H., Barkeel-Oteo, A., Coutts, A., Song, S., & Ventevogel, P. (2015). *Culture, context and the mental health and psychsocial wellbeing of Syrians: a review for mental health and psychosocial support staff working with Syrian effected by armed conflict*. Retrieved from Geneva:

James, A. C., James, G., Cowdrey, F. A., Soler, A., & Choke, A. (2015). Cognitive behavioural therapy for anxiety disorders in children and adolescents. *Cochrane Database Syst Rev, 2*, CD004690. doi:10.1002/14651858.CD004690.pub4

Jellinek, M. S., Murphy, J. M., Little, M., Pagano, M. E., Comer, D. M., & Kelleher, K. J. (1999). Use of the Pediatric Symptom Checklist to screen for psychosocial problems in pediatric primary care: a national feasibility study. *Arch Pediatr Adolesc Med, 153*(3), 254-260.

Johnson, J. G., Harris, E. S., Spitzer, R. L., & Williams, J. B. (2002). The patient health questionnaire for adolescents: validation of an instrument for the assessment of mental disorders among adolescent primary care patients. *J Adolesc Health, 30*(3), 196-204. doi:S1054139X01003330 [pii]

Jordans, M. J., Komproe, I. H., Tol, W. A., & De Jong, J. T. (2009). Screening for psychosocial distress amongst war-affected children: cross-cultural construct validity of the CPDS. *J Child Psychol Psychiatry, 50*(4), 514-523. doi:10.1111/j.1469-7610.2008.02028.x

Kessler, R. C., Andrews, G., Colpe, L. J., Hiripi, E., Mroczek, D. K., Normand, S. L. T., . . . Zaslavsky, A. M. (2002). Short screening scales to monitor population prevalences and trends in non-specific psychological distress. *Psychological Medicine, 32*(6), 959-976.

Mollica, R. F., Caspi Yavin, Y., Bollini, P., Truong, T., & et al. (1992). The Harvard Trauma Questionnaire: Validating a cross-cultural instrument for measuring torture, trauma, and posttraumatic stress disorder in Indochinese refugees. *Journal of Nervous and Mental Disease, 180*(2), 111-116.

Perrin, S., Meiser-Stedman, R., & Smith, P. (2005). The Children's Revised Impact of Event Scale (CRIES): Validity as a screening instrument for PTSD. *Behavioural and Cognitive Psychotherapy, 33*(4), 487-498.

Rahman, A., Hamdani, S. U., Awan, N. R., Bryant, R. A., Dawson, K. S., Khan, M. F., . . . van Ommeren, M. (2016). Effect of a Multicomponent Behavioral Intervention in Adults Impaired by Psychological Distress in a Conflict-Affected Area of Pakistan: A Randomized Clinical Trial. *JAMA, 316*(24), 2609-2617. doi:10.1001/jama.2016.17165

Refugees, U. N. H. C. f. (2015). 2015 UNHCR country operations profile - Syrian Arab Republic. Retrieved from http://www.unhcr.org/pages/49e486a76.html

Shelton, K. K., Frick, P.J., & Wootton, J.M. (1996). Assessment of parenting practices in families of elementary school-age children. *J Clin Child Psychol, 25*(3), 317-329.

Steel, Z., Chey, T., Silove, D., Marnane, C., Bryant, R. A., & van Ommeren, M. (2009). Association of torture and other potentially traumatic events with mental health outcomes among populations exposed to mass conflict and displacement: a systematic review and meta-analysis. *JAMA, 302*(5), 537-549. doi:302/5/537 [pii]10.1001/jama.2009.1132

Tennant, R., Hiller, L., Fishwick, R., Platt, S., Joseph, S., Weich, S., . . . Stewart-Brown, S. (2007). The Warwick-Edinburgh Mental Well-being Scale (WEMWBS): development and UK validation. *Health Qual Life Outcomes, 5*, 63. doi:10.1186/1477-7525-5-63

Thabet, A. A., Abed, Y., & Vostanis, P. (2004). Comorbidity of PTSD and depression among refugee children during war conflict. *J Child Psychol Psychiatry, 45*(3), 533-542.

van Ommeren, M., Saxena, S., & Saraceno, B. (2005). Mental and social health during and after acute emergencies: emerging consensus? *Bull World Health Organ, 83*(1), 71-75.

World Health Organisation. (2014). Health for the world’s adolescents. A second chance in the second decade. Geneva, Switzerland. [cited 2017 May 8]. Available from:<http://apps.who.int/adolescent/second-decade/files/1612_MNCAH_HWA_Executive_Summary.pdf>

**Data Analysis Plan**

Participant characteristics and potential differences between treatment completers and those who dropped out prior to the intervention period will be analyzed using planned *t* tests for continuous measures and chi-square statistics for categorical variables; for analyses of participant characteristic and differences between those who were and were not retained at follow-up, a adjusted Bonferroni adjustment to accommodate multiple comparisons.

The primary analyses of outcome measures will focus on intent-to-treat analyses. Hierarchical linear models (HLM) will be used to study differential effects of each treatment condition because this method allows the number of observations to vary between participants, which handles missing data by calculating estimates of trajectories using maximum likelihood estimation. Fixed (intervention, time of assessment) effects and their interactions will be included in the unstructured models, which provides an index of the relative effects of the treatments; time of assessment will include the baseline, posttreatment, and 3-month follow-up. Fixed effects will be tested for intervention condition and time of assessment. Fixed effects parameters will be tested with the Wald test (t-test, *p* <.05, two-sided) and 95% confidence intervals. Analyses will focus on the primary (Pediatric Symptom Checklist subscale and total scores) and secondary (Children’s Revised Impact of Events Scale, Warwick Edinburgh Mental Wellbeing Scale, Psychological Sense of School Membership, functioning, as well as caregivers’ K6 and Alabama Parenting Questionnaire scores) outcomes, with the primary outcome point being the 3-month follow-up.

To determine the reliability of this approach, we will also repeat these analyses for participants who complete the 3-month assessment. We will also conduct secondary analyses on the participants who complete the 3-month follow-up. To determine the efficacy of the EASE intervention on adolescents with internalizing problems, we will conduct secondary analyses on adolescents who score ≥5 on the PSC internalizing subscale (the recommended cut-off for identifying significant internalizing problems). We will also conduct secondary analyses to determine if the findings are influenced by exposure to prior traumatic events by repeating the primary analyses and using total scores on the traumatic events checklist as a covariate.

To determine the potential impact of EASE on parenting behaviour and adolescents’ mental health, a mediation analysis will be conducted as an exploratory secondary analysis. Specifically, separate mediation models will be conducted on participants who complete the 3-month assessment, and will focus on the extent to which the EASE and control conditions have direct and indirect effects on changes in the APQ parenting subscale scores (from baseline to 3-month follow-up), and changes in the internalizing, externalizing, and attentional problems in adolescents (from baseline to 3-month follow-up).

**Outline of EASE Program**

| **Session** | **Content** |
| --- | --- |
| **Youth Sessions** | |
| 1: Understanding my Feelings | - Welcome and group guidelines - Introduction to the storybook - Psychoeducation: understanding my feelings and identifying personal feelings |
| 2: Calming my Body | - Review home practice - Education about feelings and my body - Calming my body strategy (slow breathing) |
| 3: Changing my Actions 1 | - Review home practice - Education about feelings and actions - Changing my actions strategy |
| 4: Changing my Actions 2 | - Review home practice - Continue changing my actions strategy |
| 5: Managing my Problems 1 | - Review home practice - Understanding common problems (in young adolescents) - Managing my problems strategy (problem solving) |
| 6: Managing my Problems 2 | - Review home practice - Continue managing my problems strategy |
| 7: Brighter Futures | - Review home practice - Education about relapse prevention - Closing ceremony/activity |
| **Caregiver Sessions** | |
| 1: Understanding Sadness, Worry and Stress | - Welcome and group guidelines - Appreciating caregiver strengths - Psychoeducation: common signs of sadness, worry and stress in young adolescents - Review EASE youth sessions - Responding to a child’s feelings through active listening and slow breathing strategy - Quality time with children |
| 2: Boosting Confidence | - Review home practice - Boosting confidence in young adolescents - Appreciating children’s strengths - Praise - Alternatives to physical punishment |
| 3: Caregiver Self-care and Brighter Futures | - Review home practice - Caregiver challenges and self-care - Review caregiver sessions - Education about relapse prevention |

**Data Sharing Statement**

**Data**

**Data available**: Yes

**Data types**: Data dictionary How to access data: Request sent to Richard Bryant [r.bryant@unsw.edu.au](mailto:r.bryant@unsw.edu.au)

**When available**: With publication.

**Supporting Documents**

**Document types**: None

**Additional Information**

**Who can access the data**: Researchers whose proposed use of the data has been approved.

**Types of analyses**: For qualified researchers, to better understand what we did and to suggest additional analyses.

**Mechanisms of data availability**: The data dictionary can be made available freely. Depending on current or planned analyses of the research team, data will be made available or the authors may collaborate with other research teams on analyses with a signed research agreement.
