# Supplementary material for: Effectiveness of a brief group behavioural intervention on psychological distress in young adolescent Syrian refugees: A randomised controlled trial
Source: PLoS Med. 2022 Aug 12;19(8):e1004046. doi: 10.1371/journal.pmed.1004046 (PMC9374250; doi:10.1371/journal.pmed.1004046)
Supplement: S1 File — (PDF) [file pmed.1004046.s004.pdf]

WHO ERC
Review Summary

Protocol ID: ERC.0003012
Protocol Title: Effectiveness of Group Psychological Help for Young Adolescents Impaired by Distress in Communities Exposed to Adversity
Version: 4.4 **Dated:** 10/05/2019
WHO Responsible Staff Member: Aiysha MALIK
Responsible Unit: WHO/HQ/NMH/MSD

This project obtained approval by the ERC on 13.05.2019. The complete documentation requesting approval of amendments was submitted to the Secretariat on 31/07/2019. The outcome of review is provided below.

1. Among the screening tools to be applied to the study population , the Paediatric Symptoms Checklist 17 is to be used. Scoring 12+ is an inclusion criterion as per the approved protocol. The team is proposing to use the international standard of 15 and not the proposed of 12 as the cut-off. The reason alluded by the study team is that "Although the validation study was conducted with an ostensibly similar population of adolescent Syrians, it was decided that data from this group may not reflect the Syrian refugee population in Amman who are living in urban settings, as compared to informal settlements in Lebanon".

It was considered that the change proposed will not alter the risk:benefit ratio for participants.
Protocol **Version 4.5 Dated 31.07.2019** is approved as submitted.

Chairperson .... for

Name: Leslie Olson

Date..06/08/2019
