# Supplementary material for: Effectiveness of a brief group behavioural intervention on psychological distress in young adolescent Syrian refugees: A randomised controlled trial
Source: PLoS Med. 2022 Aug 12;19(8):e1004046. doi: 10.1371/journal.pmed.1004046 (PMC9374250; doi:10.1371/journal.pmed.1004046)
Supplement: S2 File — (PDF) [file pmed.1004046.s005.pdf]

بسم الله الرحمن الرحيم

وزارة الصحة

الرقم **م ب ا / لجنة اخلاقيات /** ١٩١١٢

التاريخ

الموافق [illegible]

مديرتطوير الموارد البشرية

تحيه طيبه وبعد ،،،

اشاره لكتابكم رقم تطوير /خطط / 10276 تاريخ2018/12/5 بخصوص البحث المقدم من قبل معهد العناية بصحة الاسرة وبالتعاون مع جامعة University of New South Wales(UNSW)

ارفق بطيه قرار لجنة اخلاقيات البحث العلمي والمتضمن الموافقة على إجراء البحث العائد للمذكورة أعلاه .

للتكرم بالاطلاع واجراءاتكم لطفا .

واقبلوا الاحترام

مدير إدارة مستشفيات البشير

الدكتور محمود سليمان زريقات

٨٩٥٠٤

بسم الله الرحمن الرحيم

وزارة الصحة

الرقم CODE : MOH REC 1800170

التاريخ ....

الموافق ....

قرار لجنة اخلاقيات البحث العلمي

إجتمعت لجنة اخلاقيات البحث العلمي بتاريخ12/4 / 2018 لمناقشة و دراسة البحث العلمي المقدم من قبل معهد العناية بصحة الأسرة وبالتعاون مع جامعة University of New South Wales (UNSW) بعنوان :

((دراسة فعالية برنامج التدخل النفسي الجمعي لدى مجموعة من الشباب المراهقين الذين يعانون من الضغوطات في المجتمعات المعرضة للأزمات))

وعليه تم التوقيع من قبل اعضاء اللجنه حسب الاصول .

عضو اللجنة
مدير مستشفى الباطني والاشعة والامراض الجلدية
الدكتور/ بشارة بقاعين

عضو اللجنة
مدير مستشفى النسائية والاطفال
الدكتور/ عصام الخوالدة

عضو اللجنة
المدير الطبي
الدكتور/ جمال حمدان

عضو اللجنة
مدير التمريض
الدكتور/ هاني القضاة

عضو اللجنة
رئيس وحدة الجودة
الدكتور/ سلامة الرجوب

مقرر اللجنة
منسق الجودة
السيد/ عيسى [illegible]

AL - BASHIR HOSPITAL
ETHICS COMMITTEE

رئيس اللجنه
مدير إدارة مستشفيات البشير

الدكتور محمود سليمان زريقات
